# In vivo methods for imaging blood–brain barrier function and dysfunction

**DOI:** 10.1007/s00259-022-05997-1

**Published:** 2022-11-28

**Authors:** William James Harris, Marie-Claude Asselin, Rainer Hinz, Laura Michelle Parkes, Stuart Allan, Ingo Schiessl, Herve Boutin, Ben Robert Dickie

**Affiliations:** 1 Geoffrey Jefferson Brain Research Centre, Manchester Academic Health Science Centre, Northern Care Alliance & University of Manchester, Manchester, UK; 2 Division of Neuroscience, School of Biological Sciences, Faculty of Biology, Medicine and Health, University of Manchester, M13 9PL Manchester, UK; 3 Division of Informatics, Imaging and Data Sciences, School of Health Sciences, University of Manchester, Manchester, UK; 4 Wolfson Molecular Imaging Centre, University of Manchester, Manchester, UK

**Keywords:** MRI, PET, Imaging, BBB, Dementia, Stroke, Alzheimer’s disease, Neuroimaging, Metabolic imaging

## Abstract

The blood–brain barrier (BBB) is the interface between the central nervous system and systemic circulation. It tightly regulates what enters and is removed from the brain parenchyma and is fundamental in maintaining brain homeostasis. Increasingly, the BBB is recognised as having a significant role in numerous neurological disorders, ranging from acute disorders (traumatic brain injury, stroke, seizures) to chronic neurodegeneration (Alzheimer’s disease, vascular dementia, small vessel disease). Numerous approaches have been developed to study the BBB in vitro, in vivo, and ex vivo. The complex multicellular structure and effects of disease are difficult to recreate accurately in vitro, and functional aspects of the BBB cannot be easily studied ex vivo. As such, the value of in vivo methods to study the intact BBB cannot be overstated. This review discusses the structure and function of the BBB and how these are affected in diseases. It then discusses in depth several established and novel methods for imaging the BBB in vivo, with a focus on MRI, nuclear imaging, and high-resolution intravital fluorescence microscopy.

## Background

The concept of a blood–brain barrier (BBB) first arose in the early twentieth century. Early studies showed that peripherally administered water-soluble dyes or toxic agents failed to stain or act in the central nervous system (CNS) or cerebrospinal fluid (CSF), whereas dyes injected into the CSF did stain brain parenchyma [1]. Since then, knowledge of the structure and function of the BBB, and its role in neuropathology, has expanded dramatically. The vertebrate BBB is a complex, heterogeneous multicellular structure, which separates the CNS from systemic circulation. It protects the delicate homeostasis of the CNS against blood-borne neurotoxic and inflammatory threats, contributes to clearance of metabolic by-products from the brain, regulates and maintains a precisely configured extracellular matrix, and mediates communication between the CNS and periphery through recruitment of immune cells and transport of soluble factors [2, 3].

BBB dysfunction plays a central role in many *acute* brain disorders including ischemic and haemorrhagic stroke, traumatic brain injury, cerebral malaria, and other central and systemic infections [4, 5]. In these conditions, blood–brain barrier damage leads to influx of blood products into the brain parenchyma, causing oedema and neurotoxicity. Subtler BBB dysfunction is also increasingly recognised as a key hallmark of *chronic* neurodegenerative disorders including AD, cerebral small vessel disease, and multiple sclerosis, and is thought to directly contribute to cognitive impairment [6–8]. Developing our understanding of the healthy and dysfunctional BBB will help to characterise, diagnose, and potentially treat such diseases [9].

In vitro models of the BBB have advanced dramatically in recent years – they can now incorporate numerous cell types in three-dimensional cultures with integrated imaging and electrophysiology [2, 10, 11]. However, whilst these models can be useful in probing certain specific aspects of dysfunction and assessing permeability of molecules of interest, they do not completely recreate the physiological environment, which limits the translatability of such studies. The complexity of the BBB necessitates in vivo studies to preserve its intricate anatomy and physiology and to truly understand pathological processes in disease. This review summarises the key established and prospective imaging techniques for probing BBB dysfunction in vivo in rodents and man.

## BBB structure and function

**Fig. 1:**
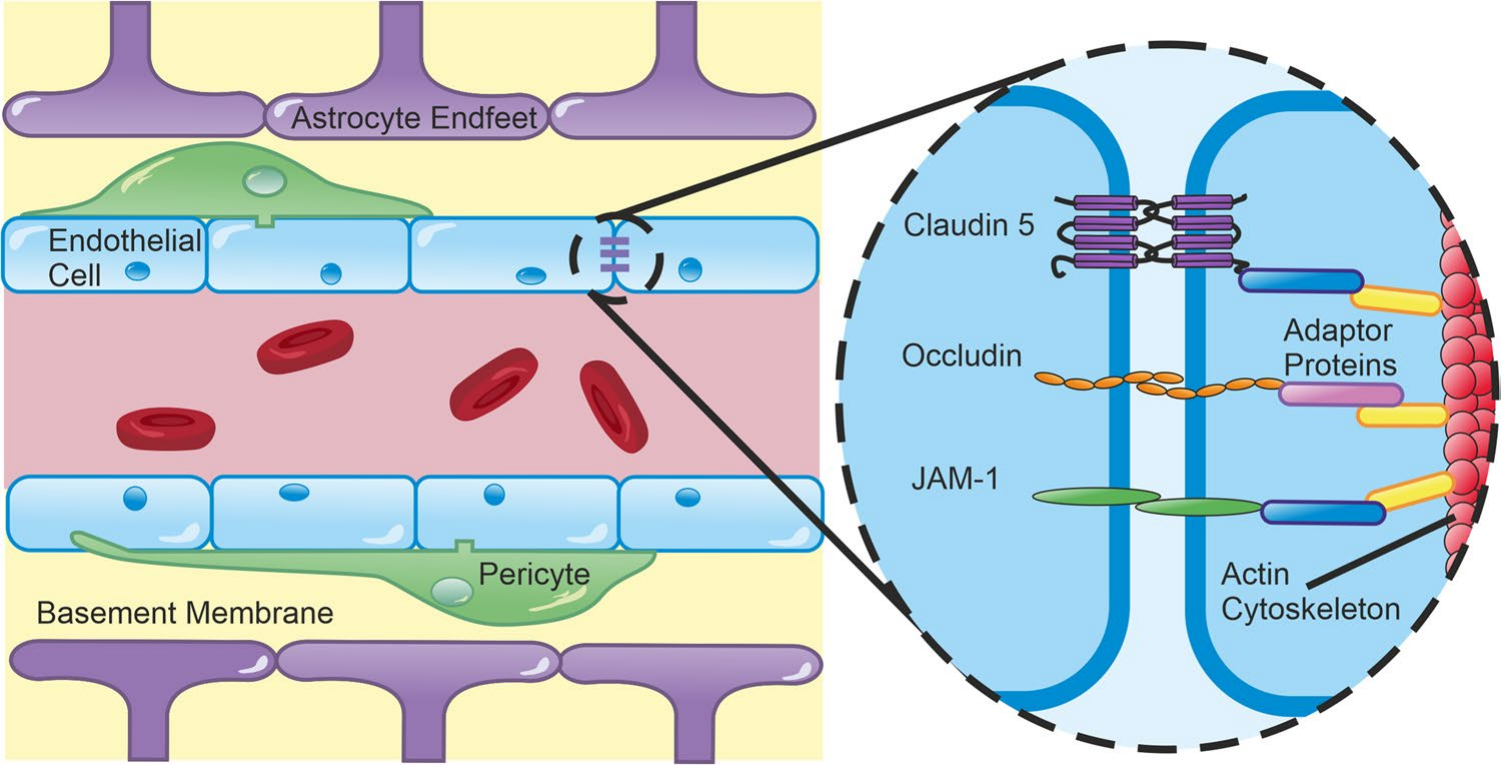
Structural Elements of the BBB. Endothelial cells are the principal component of the BBB, expressing an array of tight junctions, adherens junctions, and junctional adhesion molecules which restrict large molecules from diffusing between cells. These proteins are tethered to the actin cytoskeleton by adaptor proteins, such as ZO-1. Pericytes extend processes along and around vessels. These physically attach to endothelial cells via peg-and-socket junctions, which contribute to BBB formation and maintenance and may also actively modulate microvascular tone. Astrocytes extend endfeet to wrap cerebral vasculature. These form the glia limitans, a key element in the neurovascular unit, mediating neural control of regional blood supply and transport of a wide array of molecules and ions between circulation and neurones. The basement membrane is formed of proteins, such as laminins and collagen-IV, which are secreted by endothelium, pericytes, and astrocytes. The basement membrane is essential for BBB maintenance and is the rate-limiting step in leukocyte extravasation

The barrier properties of the BBB are stringent enough to restrict > 98% of small molecules and passive diffusion of all large molecules. The small molecules which are able to cross are those which satisfy Lipinski’s Rule of 5 [12, 13]. That is, they must be smaller than 500 Da, have fewer than five hydrogen bond donors and ten hydrogen bond acceptors, and an octanol–water partition coefficient less than or equal to 5. Certain large or polar molecules can cross the healthy BBB, although this is tightly regulated by specific receptor/transporter-mediated processes. Complex interactions between numerous cell types (Fig. 1) and molecular mediators are responsible for maintaining these properties in health, as well as modulating them in inflammatory and pathological conditions. To appreciate the value of in vivo studies—and why it is so challenging to produce physiologically accurate in vitro models—it is important to understand this complexity.

### Endothelial cells

Brain endothelial cells (BECs) are typically considered the principal component of the BBB, adapted to limit transport across (transcellular) and between cells (paracellular). BECs can be distinguished from their peripheral equivalents by their lack of fenestrae and pinocytic vesicles, which result in limited transcellular transport. They also express proteins at intra- and inter-endothelial cell borders, which impede paracellular transport. These proteins fall into three main classes: tight junctions (TJs, e.g. claudins), adherens junctions (AJs, e.g. cadherins) and cellular adhesion molecules (CAMs, e.g. JAM1, PECAM-1, ICAM-1) [14, 15], which obstruct molecules larger than 500 Da (Fig. 1) [13]. These junctional proteins also confer polarity to BECs, delineating the border between the apical (lumen-facing) and basolateral (tissue-facing) surfaces by restricting the diffusion of membrane proteins between the surfaces. The most highly expressed TJ protein at the BBB is claudin-5, but other claudins and TJ molecules, such as occludin, are important too. They are anchored to the actin cytoskeleton via adaptor proteins, such as ZO-1, ZO-2, ZO-3, and catenins. Dynamic remodelling of these complexes is involved in adaptive barrier functions, which facilitate the extravasation of circulating leukocytes [16–19]. BECs also express an anionic gel-like layer known as the glycocalyx, which extends into the lumen from their apical surface and is comprised of glycoproteins (e.g. syndecans), glycosaminoglycans (e.g. chondroitin/heparin sulfates), and glycolipids. The functions of the glycocalyx are still being elucidated, but it is believed to directly regulate the ability of circulating cells and molecules to access the BBB and it contributes to mechano-transduction of shear stress, which is necessary for junctional integrity [20, 21].

Disruption to these structures impairs the barrier function of the BBB. For example, by manipulating the amount of claudin-5 expression using knockout mice and adenovirus transfection-mediated claudin-5 knock-in mice, it has been shown to dose-dependently restrict the large (340 kDa) plasma protein, fibrinogen, from crossing the BBB [22]. This demonstrates the efficacy of TJs in preventing large-molecule paracellular diffusion. Interestingly, the prevalence of schizophrenia is higher in patients with 22q11 deletion syndrome, a disorder that reduces claudin-5 expression, highlighting the clinical importance of TJs in BBB function [22]. Leakage of endogenous molecules, such as fibrinogen and albumin, is indicative of severe BBB impairment and is well-documented in *post mortem* studies and serum measurements from neurodegenerative disease patients and animal models [23–25]. BBB permeability to smaller molecules (e.g. gadolinium-based MRI contrast agents, or water) is thought to be enhanced earlier during disease progression [26–28]. The early onset of such dysfunction has increased the popularity of the vascular theory, and vascular two-hit hypothesis of dementia, in which vascular dysfunction precedes and drives neuropathology [29, 30]. Improving the detection of subtle leakage of small molecules will enable the study of early BBB changes in vivo to determine when and where they occur, and to track the effects of therapeutics that aim to target the restoration of BBB function. Phosphorylation and translocation of TJs are also central to the development of vasogenic oedema following stroke and traumatic brain injury, in which increased BBB permeability allows plasma proteins and subsequent osmotic water movement into the brain, increasing intracranial pressure and neurodegeneration [31].

**Fig. 2:**
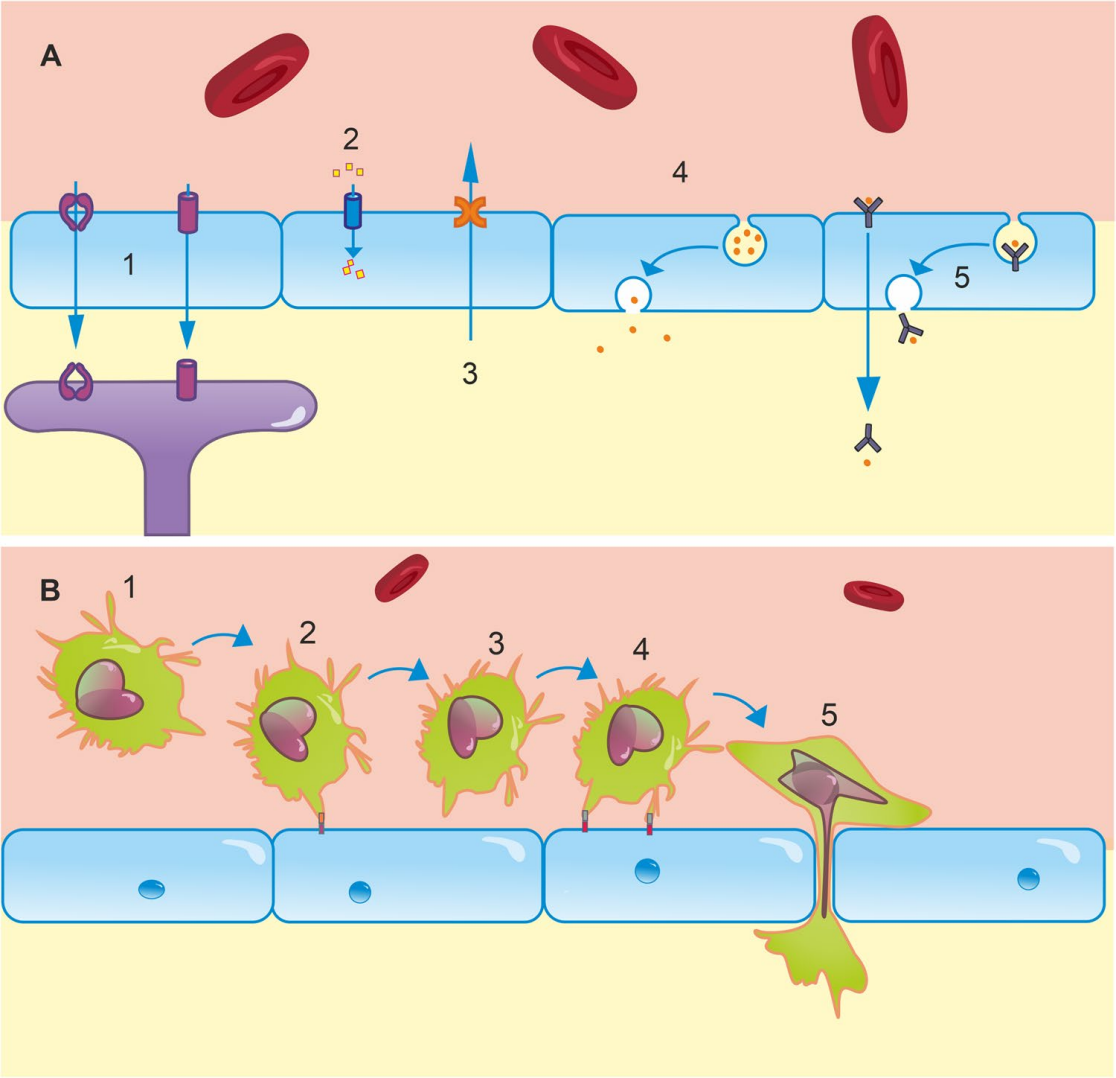
Transport mechanisms at the BBB. **A** (1) An array of transporters are expressed for a number of molecules in endothelial cells. For example, GLUT1 facilitates glucose transport from the blood to the brain down its concentration gradient. Astrocyte endfeet also express a variety of these, including a 45-kDa isoform of GLUT1, and AQP4, which regulates water uptake. (2) Ion channels in the endothelium regulate the transport of numerous ions including Na^+^, K^+^, Cl^−^, HCO3^−^, and Ca.^2+^. These support neural function and dysregulation contributes to pathologies including oedema. (3) The BBB is also fundamental in clearance of neurotoxic compounds via efflux transporters, such as P-gp and LRP1. P-gp and LRP1 dysfunction has been implicated in amyloid burden in AD. (4) Brain endothelial cells are capable of bulk transport via lipid raft invaginations known as caveolae, which play a key role in focused ultrasound-enhanced BBB permeability [32]. (5) Receptor-mediated transcytosis is a final mechanism of transport, typically responsible for transporting peptides and proteins, such as insulin, into the brain. **B** (1) At resting state, leukocytes circulate in blood and are excluded from the brain by the BBB. To enter the brain, a complex series of molecular interactions must occur [33]. (2) Rolling: endothelial receptors, e.g. E- and P-selectin, are upregulated in inflammatory states. Leukocyte plasma membrane glycoproteins bind to these, which allows them to roll along the endothelium to the site of inflammation [38]. Due to significantly reduced blood flow in the postcapillary venules of inflamed regions, these interactions are most likely to occur in postcapillary venules, rather than at other levels of the arteriovenous axis. (3) Leukocyte activation: Rolling brings leukocytes into close proximity with other inflammatory agents on the luminal surface of the endothelium. These agents activate G protein-coupled receptors, initiating intracellular cascades in leukocytes, which activate integrins expressed on leukocyte plasma membranes [33]. (4) Adhesion: integrins typically bind to extracellular matrix proteins secreted by endothelial cells, such as ICAM-1, VCAM-1, and PECAM-1 [39]. This tight binding halts rolling. Leukocytes crawl along the endothelium to cell borders either with or against the direction of blood flow. (5) Diapedesis: leukocytes cross the BBB between or through endothelial cells. This is dependent on homophilic interactions between receptors on leukocytes and those on endothelial cells, basement membrane, and pericytes [33]

Paracellular diffusion is just one means of trans-BBB transport. Additionally, molecules can access the CNS via receptor-mediated, carrier-mediated, or adsorptive transcytosis; ions can cross the barrier via ion pumps/channels, and an armoury of efflux pumps actively clears the CNS of toxic compounds and waste products (Fig. 2a) [15]. Adsorptive-mediated transcytosis by lipid invaginations known as caveoli also plays a role in bulk transport, predominantly of larger molecules. Caveoli appear to be important in focused ultrasound-enhanced BBB permeability to large molecules, with a key component (caveolin-1) being upregulated in sonicated mouse hippocampi, and caveolin-1 knockouts showing reduced permeability to 500 kDa dextran following sonication [32]. Finally, peripheral immune cells are able to cross the BBB. This is a multi-step process involving leukocyte adhesion to BECs, rolling, and diapedesis (Fig. 2b) [33, 34]. This may be paracellular (necessitating dynamic alterations to TJ arrangement) or transcellular and primarily occurs at the post-capillary venule in inflamed brain regions, important for the CNS inflammatory response [35, 36]. These diverse pathways create a network of regulated transport mechanisms by which the brain can extract essential nutrients (glucose, amino acids, etc.) from the blood, and extrude harmful compounds and metabolic by-products. Disturbances to any of these can destabilise CNS homeostasis, resulting in excessive accumulation of harmful substances or insufficient supply of essential nutrients. For example, increased uptake of amyloid peptides via Receptor for Advanced Glycation End-products (RAGE) and reduced clearance from the brain via the active efflux transporters of amyloid peptides, p-glycoprotein (P-gp), and LDL receptor–related protein 1 (LRP1), contribute to the amyloid burden pathognomonic of AD [37]. Elevated P-gp function in stroke and treatment-resistant epilepsy also hampers the delivery of potential therapeutics [37]. Improving methods of quantifying BBB transport will help develop a more comprehensive understanding of homeostatic challenges in diseases and may improve diagnoses.

### Pericytes

Whilst endothelial cells form the primary physical barrier, several other cell types are required to develop and maintain the BBB, as well as modulate its function. Pericytes are morphologically diverse motile cells embedded in the basement membrane throughout the cerebral microvasculature, which are capable of proliferation and migration to sites of injury and angiogenesis [40]. They extend far-reaching (~ 300 µm) processes either longitudinally or circumferentially, which physically attach to multiple BECs via peg-and-socket and gap junctions (Fig. 1) [41]. This facilitates paracrine and juxtacrine signalling, which is essential for the development and maintenance of the BBB [42–45]. In addition to maintaining BBB integrity, physical contact via peg-and-socket junctions may allow pericytes to exert direct contractile force on endothelial cells and actively modulate microvascular tone, although this remains controversial [46]. Some groups have argued that capillary pericytes rather than arteriolar smooth muscle cells are responsible for the majority of functional hyperaemia [47, 48]. This may occur specifically at post-arteriole capillary junctions, where ensheathing pericytes modulate flow into specific regions of the capillary bed by detecting extracellular K^+^ and initiating and propagating capillary dilatation from the site of stimulus to upstream vessels [49]. Contrary to these findings, alternative data derived by the same modality (high-resolution in vivo two-photon imaging) suggest that capillaries lack vasomotor responses and that smooth muscle cells on arterioles are responsible for controlling vascular tone [50]. These controversies may partly stem from ambiguity over distinctions between pericytes and smooth muscle cells. The development of more specific molecular markers and higher resolution imaging modalities will help characterise the morphology and localisation of each cell type more clearly.

The physiological significance of pericytes is highlighted by their involvement in a range of neurological disorders. Pericyte-deficient mice show clear structural abnormalities in the cerebral vasculature, associated with increased deposits of immunoglobulins (IgG) and fibrinogen [51]. This demonstrates the importance of pericytes in BBB maintenance. Vascular pericyte coverage decreases with age in C57BL/6 mice, which leads to concomitant reductions in pericyte-induced gene expression in endothelial cells and increased extravasation of plasma proteins [52], suggesting BBB impairment occurs during normal ageing. Pericytes are also implicated in disease; levels of the pericyte marker soluble platelet-derived growth factor receptor β (sPDGFRβ) are significantly elevated in CSF of cognitively impaired patients [24, 53, 54]. The presence of sPDGRFβ in CSF indicates pericyte damage, suggesting that their death or dysfunction may contribute to cognitive impairment. Moreover, exogenous and endogenous amyloid oligomers constrict capillaries near pericytes, but not arterioles and venules in human and murine brain tissue. This suggests that pericytes may be responsible for the early blood flow reductions seen in AD [55]. Pericytes are also particularly susceptible to stroke, following which a sustained pericyte contraction has been observed [48]. This may underlie the post-ischemic no-reflow phenomenon [48, 56, 57], wherein capillary blood flow is not restored after the recommencement of arterial flow.

### Astrocytes

Astrocytes are another major component of the BBB and neurovascular unit. The cells are connected in syncytium by gap junctions, which facilitates rapid signalling across large areas by calcium waves, and diffusion of metabolites and other molecules [58, 59]. They are highly polarised cells, extending perivascular endfeet which ensheath the endothelium—this forms a secondary barrier known as the *glia limitans—*and endfeet which ensheath synapses, where they modulate, receive, and directly contribute to synaptic signalling via gliotransmission [60].

Perivascular endfeet cover almost all cerebral microvasculature. As with pericytes, these are important for the formation and maintenance of endothelial TJs, but they also express dense and varied transport proteins, including aquaporin 4 (AQP4), GLUT1, and big current potassium (BK) channels [61–63]. These proteins play fundamental roles in bidirectional neurovascular coupling. For example, astrocytes can stimulate vasodilatation or constriction, dependent on the magnitude of astrocytic calcium oscillations [64]. These calcium signals can be driven by metabotropic glutamate communication with neurones [65], demonstrating the role of astrocytes in directing blood flow to areas of neuronal activity.

CBF regulation is intrinsically linked to neuronal metabolism. Astrocytes further support neuronal metabolism by storing and supplying metabolites on demand. The Astrocyte-Neurone Lactate Shuttle hypothesis proposes that astrocytes mediate the majority of activity-dependent energy supply to neurones [66]. In this model, glucose enters astrocytes via GLUT1 and is either stored as glycogen or glycolytically metabolised into lactate. Lactate derived from either glycogen or glucose can then be shuttled from astrocytes into neurones by monocarboxylate transports (MCTs) [67, 68]. Lactate can then be converted to pyruvate, which acts as a substrate in the tricarboxylic acid cycle, fuelling oxidative phosphorylation in neurones. This is regulated by the degree of neuronal activity, whereby rising extracellular concentrations of potassium [69] or glutamate [66] indicate increased action potential firing and stimulate astrocytic glycogenolysis. This represents a mechanism by which metabolically active neurones can be supplied with an energy source in an activity-dependent (efficient) manner, and by which glucose can be stored in astrocytic glycogen deposits, which act as a buffer in the event of glucose deprivation, such as in ischemia. Since its proposal, the ANLS hypothesis has remained controversial. This debate has been extensively reviewed elsewhere [70, 71] and contributing to this discussion is not the focus of this review. Glucose hypometabolism is a characteristic early pathology in Alzheimer’s and has been reliably detected by FDG-PET in the clinic and in rodent models [72–76]. If astrocytes are the major supplier of neuronal energy substrates, this suggests that astrocytes, not neurones, may be the source of this deficiency [77, 78].

Astrocytes are not just central to neuronal nutrient supply; they also play a key role in clearing waste products from the brain. AQP4 contributes to the mixing of perivascular CSF with interstitial fluid. This is necessary for a recently identified bulk clearance mechanism, known as the glymphatic system, which removes toxic compounds including amyloid and tau peptides from the brain, as reviewed by Rasmussen et al. [79]. Alterations in the expression and localisation of AQP4 can profoundly affect glymphatic clearance. Loss of astrocytic AQP4 polarity, for example, is correlated with cognitive decline, Braak stage, and amyloid burden in AD [80]. AQP4 is also implicated in the pathophysiology of stroke and traumatic brain injury. In healthy tissue, ion channels and AQP4 maintain water homeostasis, which is essential to maintain cell/tissue volume. In ischemic stroke, however, cellular oedema is observed rapidly, followed by vasogenic oedema in the subacute phase (24–48 h) [81]. Preclinical models have shown that AQP4 expression directly correlates with cytotoxic oedema [82], and this can be driven by the translocation of AQP4 to the plasma membrane [83]. AQP4 knockouts show marked reductions in cytotoxic oedema [62] and water exchange across the BBB [84]. In contrast, AQP4 knockouts have also demonstrated exaggerated swelling in vasogenic oedema models [85], whereby luminal water crosses the BBB and builds up in the CNS. This highlights a complex relationship between astrocytes, AQP4, and the development and resolution of oedema.

### Basement membrane

The BBB is encased by a basement membrane, a network of extracellular matrix proteins secreted by endothelial cells (endothelial basement membrane) and pericytes/astrocytes (parenchymal basement membrane) [86–88]. The membrane is a network consisting primarily of diverse isoforms within the families of laminins, collagen IV, nidogens, and heparan sulfate proteoglycans [89]. These are molecularly and functionally distinct layers [90, 91] which add an extra barrier, and also support and facilitate interactions between the cells of the BBB [4, 92]. This barrier is considered the rate-limiting step in leukocyte diapedesis [93], indicating its importance in CNS immune privilege. Leukocytes pass the barrier by secreting matrix metalloproteinases to degrade the membrane (this takes around 30 min, compared with 3–4 min to cross the endothelial monolayer [94]). These infiltrating cells cross the BBB at sites dependent on which basement membrane laminins are expressed. T lymphocytes, for example, cross in areas of low laminin 511 expression, whilst neutrophils and monocytes can cross in areas with either laminin 411 or laminin 511 expression [91, 94].

In addition to its role as a physical barrier, the basement membrane anchors cellular components to the barrier with cell-type-specific integrin and dystroglycan receptor interactions. These interactions are important for BBB function; the extent of collagen IV interaction with endothelial integrin receptors is correlated with claudin 5 expression, for example [95]. Furthermore, laminin 511 has been shown to promote VE-cadherin expression at endothelial junctions and increase transendothelial electrical resistance, a measure of paracellular integrity [94]. This is thought to be a specific interaction, as the study did not find this effect with laminin 411 or non-endothelial laminins. Additionally, astrocyte endfeet express integrin α2, which interacts with endothelial laminin to promote a BBB-protective phenotype of pericytes, AQP4 expression in astrocyte endfeet and inter-endothelial TJ formation [96].

The basement membrane is difficult to study for two reasons. First, many components of the membrane are widely expressed, and a number of knockout models prevent development past the embryonic stage, as reviewed by Thomsen et al. [4] Secondly, the complex structural assembly of the different molecular components—and the variability of this composition along the arteriovenous axis and between tissues—prevents accurate recreation of the complete basement membrane in vitro. For these reasons, and the variety of specific molecular interactions detailed in the previous paragraph, basement membrane research benefits greatly from studying the intact BBB.

Alterations in the composition of the basement membrane are observed in a range of diseases including diabetes, and AD [4]. Cerebral amyloid angiopathy, a major vascular component of AD, is associated with significant amyloid deposition in the basement membrane, as well as vessel walls [97]. Furthermore, basement membrane thickening is observed in AD, hypertension, small vessel disease, and diabetes [98–101]. In contrast, the upregulation of proteases and inflammatory cytokines in stroke contributes to the degradation of basement membrane components, including collagen IV, agrin, laminins, and fibronectin [102–105].

### Immune cells, microglia, and peripheral factors

The BBB confers immune privilege to the CNS; i.e., peripheral immune cells are predominantly excluded. However, the BBB is profoundly affected by inflammatory mediators, such as cytokines and oxidative species, which are secreted by the pro-inflammatory ‘activated’ microglia and astrocytes in the brain, as well as infiltrating immune cells. As such, inflammatory mediators, immune cells, and glia can dictate whether the BBB is intact or ‘leaky’ [2, 3, 106]. Additionally, glial and immune cells secret matrix metalloproteinases (MMPs), which are necessary for leukocyte infiltration and degrade both paracellular junctions and basement membrane to increase BBB permeability in neurodegeneration and ischemia [107, 108]. In acute inflammation, this modulation allows peripheral monocytes to invade and tackle CNS pathogens and remove harmful compounds. However, persistent inflammation is believed to initiate a self-perpetuating increase in BBB permeability and this may contribute to the pathogenesis and symptoms of neurodegenerative diseases [106]. This is exemplified by the duality of microglial behaviour in acute and chronic inflammation. Acute systemic inflammation induced by LPS injection promotes microglial migration to the BBB, where they express claudin 5 and extend processes through the basement membrane to contact endothelium [109]. This appears to support the BBB, partially mitigating the impairment caused by the inflammation. With continued daily LPS injections, however, conditional microglia knockout mice and minocycline-treated mice show reduced BBB permeability, suggesting that in chronic inflammation activated microglia are detrimental to the barrier.

### Heterogeneity of the BBB

CNS barriers are highly heterogeneous between brain regions. For example, the hippocampus is more vulnerable to ageing- and hypertension-associated BBB leakage [24, 110], the distribution and phenotype of pericytes varies with cortical depth [111], and the location of stroke has a significant effect on leukocyte infiltration [112].

In addition to regional variations, BBB structure and function vary according to the level of the vascular tree. For example, leukocyte infiltration preferentially occurs at post-capillary venules [113]. There is also significant variation in the basement membrane thickness and laminin composition between vessel types [4]. Furthermore, endothelial cells display *continuous* transcriptomic changes along the axis, whereas there are *discrete* transcriptional/morphological classes of mural cells [111, 114, 115].

A number of CNS barriers exist. The blood-CSF barrier is a functionally and structurally distinct barrier in the choroid plexus and circumventricular organs [116, 117], and the blood-spinal cord barrier is another distinct barrier encasing spinal vessels. These heterogeneities are frequently overlooked and the barriers are sometimes incorrectly collectively referred to as the BBB [114].

Macroscopic imaging techniques, such as MRI and PET, facilitate the characterisation of regional heterogeneity (i.e. differences in the BBB between regions). Microscopic techniques enable detailed visualisation of heterogeneities occurring over smaller scales (i.e. along the arterio-venous axis).

## In vivo imaging techniques of the BBB

The complexity of the BBB and the array of diseases in which several components are affected necessitates a multimodal approach to imaging these changes in vivo. An overview of these structural/functional changes is given in Table 1, alongside appropriate imaging modalities used to detect them.

**Table 1:** Brief summary of BBB transport-related pathologies and appropriate imaging modalities to investigate these

Pathology	Effect on BBB transport	Relevant diseases	In vivo imaging modality
Downregulation or structural alterations of TJs, AJs, and JAMs	Increased paracellular permeability increases free diffusion between CNS and blood. This allows harmful compounds and peptides into the CNS	AD [118], cerebral small vessel disease [119], stroke [120], epilepsy [121], peripheral and CNS infections [122], brain tumours [123], Parkinson’s disease [124], multiple sclerosis [120], Huntington’s disease [124], amyotrophic lateral sclerosis [125], schizophrenia [124], chronic pain [126], diabetes mellitus [127], psychostimulant abuse [128], traumatic brain injury [61], chronic stress [129]	DCE MRI [130–137], water-exchange MRI [28, 84, 138], PET [139–141], intravital microscopy of dextran leakage [129, 142]
Upregulation of cellular adhesion molecules	Increased leukocyte adhesion and diapedesis	Peripheral and CNS infections[122], AD [143], stroke[144], traumatic brain injury [145], multiple sclerosis [146], cardiac arrest [147]	MPIO or USPIO MRI [144, 148–157], antibody/nanobody tracers for PET/SPECT [158]
Degradation of endothelial glycocalyx	Improved access to the apical surface of the endothelium, facilitating extravasation of water (oedema) and peripheral immune cells. Also affects adsorptive transcytosis	Stroke [159], traumatic brain injury [160], infections[122], cardiac arrest [147]	Intravital microscopy [161, 162]
Degradation or thickening of basement membrane	Degradation increases rate of diapedesis, detachment of pericytes and astrocyte endfeet. thickening reduces the elasticity of vessels	AD [163], Parkinson’s disease [163], amyotrophic lateral sclerosis [163], stroke [164]	Intravital microscopy to image pericytes and astrocyte endfeet (not a direct measure of basement membrane but can indicate its integrity; direct assessment will require labelling strategies for basement membrane components) [41, 165–167]
Altered expression or function of uptake or efflux transporters	Reduced transcellular uptake of nutrients (e.g. glucose), enhanced uptake of toxic compounds (e.g. amyloid peptides), impaired clearance of toxic compounds or drug resistance	AD [168], Parkinson’s disease [37], epilepsy [121], tumours [169], Stroke [170]	Molecular MRI (glucose-sensitive MRI, deuterium metabolic imaging, etc.) [171–175] and PET/SPECT [176–180] (combined with appropriate sampling for kinetic modelling)
Leukocyte extravasation	May be important in disease resolution, but also contributes to transduction of inflammation from the periphery to the CNS and the perpetuation of inflammation within the CNS	Stroke [9], traumatic brain injury [181], multiple sclerosis [9], Infections [122], AD [39]	Intravital microscopy [182, 183], USPIO MRI [143]

### Magnetic resonance imaging

A range of magnetic resonance imaging (MRI) techniques exist to probe a variety of BBB functions. This permits investigation of BBB integrity and transport under physiological conditions and, as they are largely non-invasive, they provide a means to measure BBB function in humans. As a non-ionising modality, it is also feasible to perform longitudinal MRI studies to track disease progression in clinical and preclinical studies.

#### DCE-MRI

**Fig. 3:**
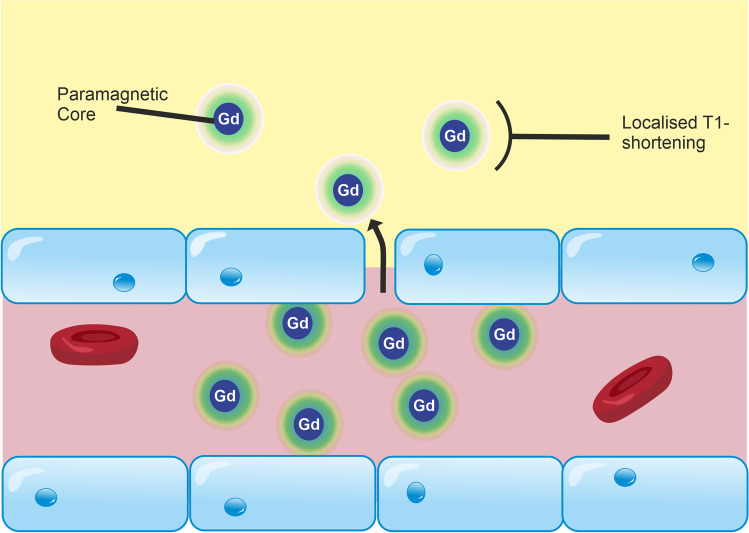
Leakage of GBCA across the BBB in DCE-MRI. DCE-MRI uses an intravenous injection of *T*_1_-shortening GBCAs. Under normal conditions, GBCA molecules are restricted from the CNS by the BBB. Inflammation reduces the junctional expression of TJ proteins in endothelial cells, which increases the size of molecules that are able to cross the BBB. As a result, GBCA molecules are able to permeate the CNS. These molecules consist of a paramagnetic core, which interacts with adjacent water molecules to increase the rate of *T*_1_ relaxation, which is detected as a signal enhancement on *T*_1_-weighted MRI. By scanning dynamically and applying kinetic models to the change in signal over time, the leakage rate of GBCA can be estimated and used as a measure of paracellular integrity

**Fig. 4:**
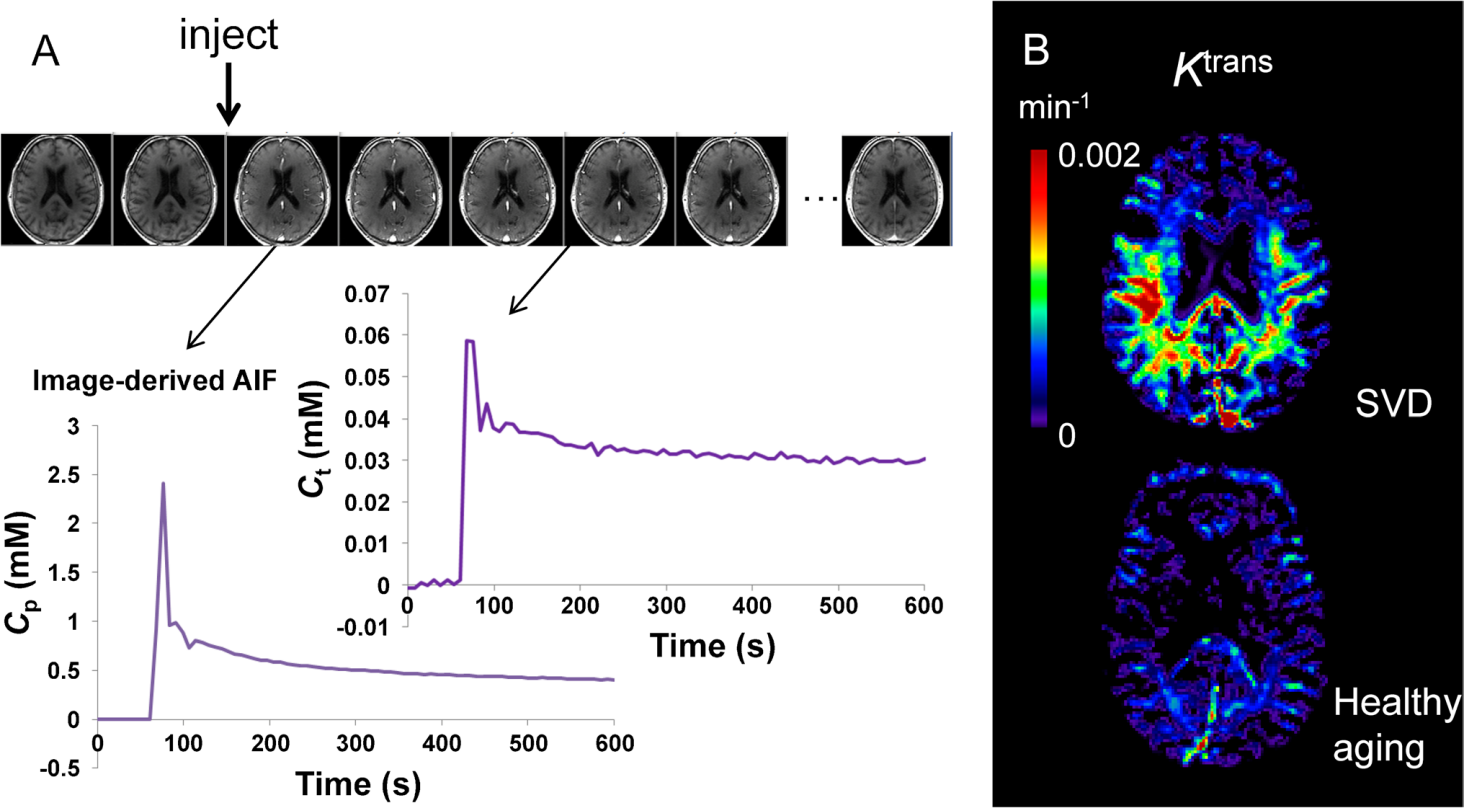
Dynamic contrast-enhanced MRI for the estimation of contrast agent leakage rate (*K*^trans^) across the BBB. **A** Gadolinium-based contrast agent is injected during dynamic collection of *T*_1_-weighted images. The contrast agent enhances the signal, first in the blood, and later in the tissue as the contrast agent leaks across the BBB. The concentration of contrast agent in the blood plasma (*C*_p_) of a feeding artery and each tissue voxel (*C*_t_) can be measured over time. A kinetic model is fitted to this data in order to calculate *K*^trans^ in each voxel of the brain. **B** Example *K*^trans^ images in a patient with small vessel disease (SVD) aged 73 years (top) and a woman with no health problems aged 68 years (bottom). Elevated *K*^trans^ can be seen in SVD, predominantly in the white matter. Note that the dynamic series of images in **A** is from the gentleman with cSVD shown in **B**. The images kindly provided by Dr Laura Parkes were taken from a study acquiring DCE-MRI data in people with Parkinson’s disease and/or cerebrovascular disease [186]

MRI-based measurements of BBB integrity are typically performed via *T*_1_-weighted dynamic contrast-enhanced (DCE)-MRI [113, 114], in which a contrast agent—typically a paramagnetic gadolinium-based contrast agent (GBCA)—is injected intravenously and leakage of the agent across the BBB is detected as a change in the *T*_1_ of brain tissue (Fig. 3). The aim of DCE-MRI assessment of the BBB is to estimate the contrast agent transfer constant, *K*^trans^, which is a quantitative measure of the rate of indicator transfer from the vascular to extravascular space. Due to the 3D macroscopic resolution of DCE-MRI, it is possible to determine region-specific *K*^trans^ values in humans and animals [24, 184]. This method has been used to demonstrate BBB leakage in cerebral small vessel disease [6] (Fig. 4), AD [24, 185], and MS [131], as well as in conditions with more severe leakage such as stroke and brain tumours.

Since each voxel of brain tissue contains both blood (approximately 5% of the voxel volume) and parenchyma, a measured arterial input function combined with kinetic models are needed to infer vascular and extravascular (leakage) contributions to the signal enhancement. To allow kinetic analysis, measured *T*_1_-timecourses are converted to GBCA concentration–time courses using the GBCA spin–lattice relaxivity factor, *r*_1_:

1 $${C}(\mathrm{t})=\frac{1}{{{r}}_{1}}\left(\frac{1}{{{T}}_{1}(\mathrm{t})}-\frac{1}{{{T}}_{10}}\right)$$

where *T*_10_is the pre-contrast spin–lattice relaxation time and *T*_1_(t) is the post-contrast *T*_1_.

**Fig. 5:**
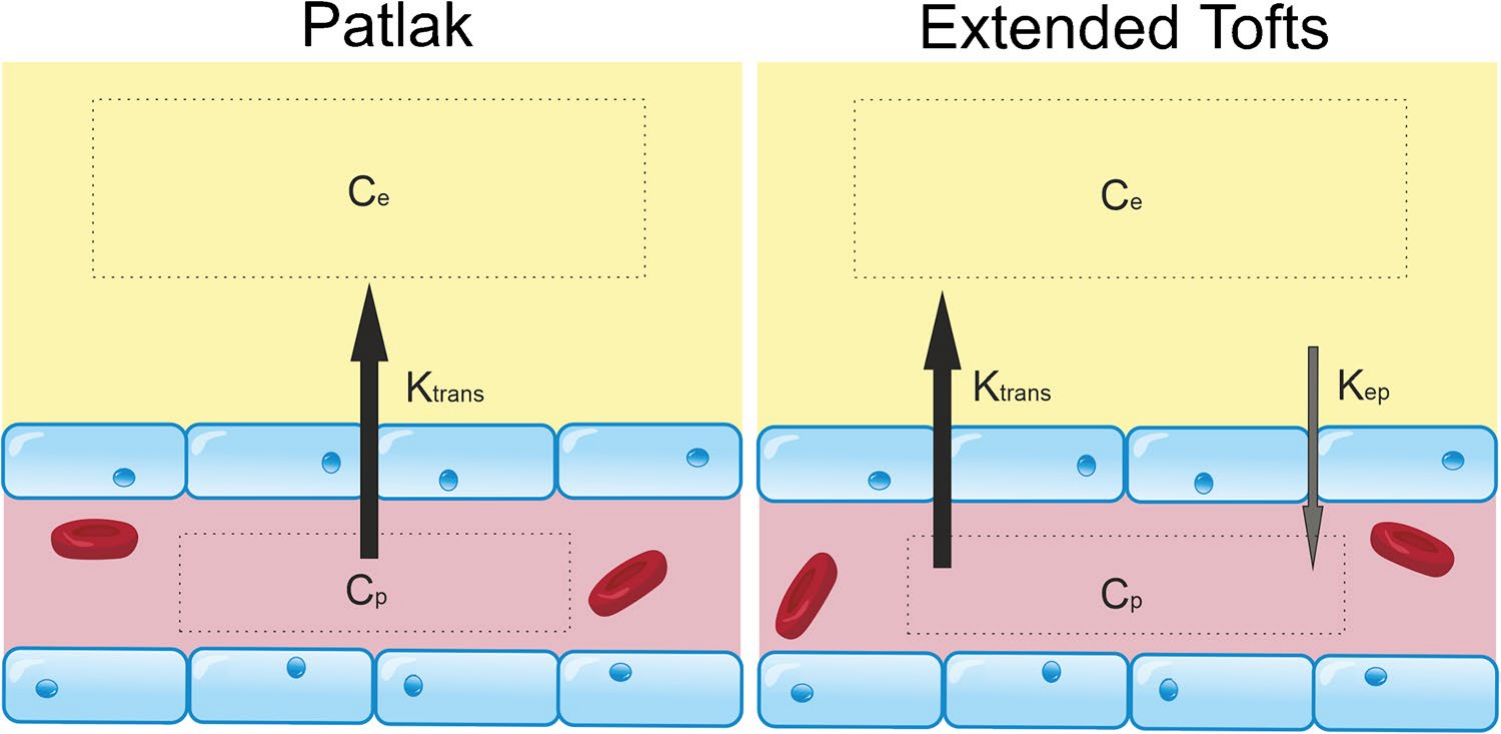
Tracer kinetic models for subtle BBB leakage. Two common models for estimating gadolinium leakage across the BBB rely on different assumptions and are appropriate in different contexts. The Patlak model assumes that backflux of the compound of interest into the blood is negligible during the timeframe of the imaging experiment, a condition that is met when leakage is very low such that the concentration of contrast agent in plasma (*C*_p_) is always in significant excess of that in tissue (*C*_e_). The extended Tofts model allows for some backflux of tracer into the blood (represented by the constant, *k*_ep_, which is equal to *K*^trans^/*v*_e_, where *v*_e_ is the extravascular extracellular volume fraction), and is considered a more suitable model in cases of more significant BBB impairment

The most appropriate kinetic model to use depends on the level of indicator leakage. In a healthy brain, and brains with subtle BBB pathology (e.g. neurodegenerative disorders), it is accepted that the Patlak model [187] (Fig. 5) is most appropriate for estimating *K*^trans^ [133, 137]. Using this model, the voxel concentration of GBCA, *C* [mM], is given by:

2 $${C}(\mathrm{t})= {{v}}_{\mathrm{p}}{{C}}_{\mathrm{p}}(\mathrm{t})+{{K}}^{\mathrm{trans}}{\int }_{0}^{\mathrm{t}}{{C}}_{\mathrm{p}}(\mathrm{t})\mathrm{\;dt}$$

where *v*_p_ [mL plasma/mL tissue] is the fractional plasma volume, *C*_p_ [mM] is the concentration of contrast agent in capillary plasma, and *K*^trans^ is the volume transfer constant (min^−1^) of contrast agent from the blood–brain.

This model assumes that:

i. The indicator has access to two compartments separated by the blood–brain barrier, namely the vascular compartment and the extravascular extracellular compartment.
ii. The indicator extravasation is permeability limited (i.e. cerebral blood flow >  > PS). Under these conditions, *K*^trans^ is approximately equal to the permeability surface area product (PS).
iii. The bolus of indicator undergoes zero dispersion between arterial and capillary blood (i.e. the GBCA concentration is equal in arteries and capillaries; *C*_p_ = *C*_a_). Under these conditions, the measured arterial input function can be directly used in Eq. 1 in place of *C*_p_ [188].
iv. That water exchange across the BBB and other cell membranes is infinitely fast relative to differences in compartmental spin–lattice relaxation rates. This assumption is valid under most conditions, but may transiently exit from these conditions during the peak plasma concentrations, or in the presence of substantial indicator leakage into tissue [27, 135].
v. That efflux of indicator back into the blood during the measurement duration is negligible, and thus, the tissue compartment can be treated as irreversible. This assumption is akin to assuming infinitely large interstitial volume, *v*_e_. It will be violated if *v*_e_ is unexpectedly small, or if *K*^trans^ is high [188].
vi. That indicator is well-mixed within each compartment.

In areas with significant BBB leakage (e.g. haemorrhage or tumour), extravasation of indicator into brain tissue will depend on both cerebral blood flow (delivery of indicator to the vascular bed), and vascular permeability (i.e. PS) [189, 190]. The effects of P and S cannot be distinguished, and thus *K*^trans^ is not a true marker of permeability—it will depend on fractional blood volume and vessel size distributions, which may contribute to inter-regional variability and may also be affected in disease states. Methods to measure microvessel diameter using MRI have been developed [191, 192], but have not yet been used in combination with measures of *K*^trans^ to derive measures of *K*^trans^ independent of vessel surface area. Alternatively, a marker of vascular leakage that is independent of blood volume can be determined by calculating the exchange rate (*k*_Gad_) by dividing *K*^trans^ by the plasma volume fraction (*v*_p_) [130]. This parameter reflects how often on average an indicator particle exchanges from blood to tissue. It is unclear how valid such an assumption is, given the Patlak model assumes negligible efflux of indicator back into the bloodstream.

When BBB impairment is more severe, the assumption of negligible efflux of tracer back into the vascular space during the measurement duration may be inaccurate. Under those conditions, the extended Tofts model, which accounts for finite efflux, may be more accurate [193, 194]. A common assumption to the Patlak and extended Tofts models is that contrast agent concentration is equivalent between arteries and capillaries. If cerebral blood flow is reduced (for example in patients with AD, or due to stroke), it is possible that the indicator bolus undergoes dispersion between the feeding artery and capillaries (*C*_p_ ≠ *C*_a_).

Arterial input functions (AIFs) are used across dynamic imaging modalities to describe the delivery of indicator to tissue as a function of time, which is required for accurate kinetic modelling. The regular sampling of arterial blood necessary to estimate this accurately is highly invasive and technically challenging. In DCE-MRI, it is possible to use image-derived methods to quantify the arterial contrast agent concentration without the need for blood testing; this method requires the image analyst to segment an image region containing arterial blood (usually a large artery), and to convert measured R_1_ time courses to contrast agent concentration using a pre-defined calibration constant called the relaxivity. However, factors such as inflow effects, and partial volume effects of the artery with surrounding tissue, and insufficient temporal resolution, make indicator concentrations in blood difficult to measure accurately [6]. For DCE-MRI in the brain, temporal resolutions of between 5-60 s are possible depending on the imaging protocol. Simulations have shown errors to be small for temporal resolutions < 60 s, which is readily achievable using modern hardware [195]. Furthermore, it is possible to acquire data using a dual-resolution approach, whereby data around the AIF peak is acquired using lower-spatial resolution and higher temporal resolution than the data after the peak (i.e. during the leakage phase), which produces robust leakage estimates [196]. Finally, it is possible to use a slower injection, which reduces the temporal resolution requirements further by smoothing out the AIF peak and reducing sensitivity to flow effects [197]. Most contrast agents are extracellular and do not cross intact cell membranes. This has implications for kinetic modelling, since only compartments accessible to the contrast agent contribute towards observed signal changes, and thus can be modelled. This means that plasma contrast agent concentrations must be calculated from whole blood AIFs. For DCE-MRI agents, rapid water exchange between plasma and erythrocytes means that plasma AIFs can be calculated by scaling the whole blood AIF by 1/(1-Hct), where Hct is the arterial haematocrit. Clearly, any error in measuring the haematocrit will lead to errors in the estimate of the plasma AIF.

Despite DCE-MRI forming the cornerstone of MRI BBB studies, there are limitations associated with the use of GBCAs. For example, they may be too large to diffuse across the BBB unless BBB breakdown is relatively severe [198, 199]. Thus, using DCE-MRI to study diseases with subtle BBB dysfunction, such as AD and small vessel disease, where the leakage is very slow, and associated MRI signal changes are of low magnitude relative to noise, is challenging. Furthermore, gadolinium leakage is paracellular and so, whilst it is a useful indicator of junctional integrity, it cannot be used to assess the function of specific transcellular transport systems.

Other factors confound DCE-MRI measurements of subtle BBB leakage, including artefacts (Gibbs ringing), scanner drift, and heterogeneity between tissues (partial volume effects), which can obscure leakage being distinguished from background noise [27, 132], or lead to misinterpretation of results. The choice of model, as discussed above, can also influence findings. Furthermore, concern has been growing regarding the unknown long-term consequences of gadolinium accumulation, which has been observed in numerous brain regions including the thalamus, substantia nigra, and red nucleus in patients with seemingly intact BBB [200]. Whilst DCE-MRI has proved very useful, these limitations suggest it may be beneficial to develop improved contrast agents or contrast-free MRI techniques.

#### Water-exchange MRI techniques for probing BBB function

To address the low sensitivity of DCE-MRI, there has been extensive work to develop methods that quantify the rate of water exchange across the BBB [27]. As a smaller molecule, it has greater BBB permeability than GBCAs, exchanging several thousand times faster than GBCAs [130]. It may, therefore, be more sensitive to subtle changes in junctional integrity. In contrast to GBCAs, water can pass across the BBB through trans-membrane proteins, ion channels [201] and AQP4 water channels located on astrocyte endfeet [84]. Changes in water permeability may therefore occur through a much wider range of mechanisms than an alteration to junctional integrity and so it has less pathological specificity than DCE-MRI. There are three main approaches for measuring BBB water-exchange: contrast-enhanced techniques [28, 202–204], arterial spin labelling (ASL) techniques [27, 138, 205–210], and more recently approaches based on double-diffusion encoding MRI [211]. All these techniques aim to detect the effects of trans-BBB exchange on either *T*_1_, *T*_2_, ADC, or any other detectable NMR property that is sufficiently different between intra- and extravascular compartments.

Contrast agent-based techniques use an intravascular indicator to preferentially shorten the *T*_1_ or *T*_2_ of blood. Water has a similar *T*_1_ relaxation rate in intra- and extravascular compartments and, due to the small size of the intravascular compartment, MRI cannot reliably detect these differences. Intravascular contrast agents are often used to shorten the *T*_1_ of blood water, increasing the difference between intra- and extravascular compartments [28, 202]. This enhances the sensitivity for detecting bi-exponential relaxation, facilitating the estimation of the blood-tissue water exchange rate (*k*_in_, also referred to as *k*_be_). In contrast to other approaches for measuring water-exchange discussed later, GBCA approaches also enable the estimation of cerebral blood volume, proving a means to also calculate the permeability surface area product, *PS*_w_. This provides a measure of the total amount of water exchanging per unit time (taking into account the contribution from blood volume), whereas *k*_in_ provides information only on the frequency at which each water molecule exchanges [27].

ASL water exchange techniques study the kinetics of tagged arterial water as it passes through the vascular tree and into tissue. *T*_o_ estimate water exchange, standard ASL can be extended and combined with *T*_2_/*T*_2_*- or diffusion-weighting, which enables label localisation (intra- or extravascular) to be determined as a function of post-label delay time. These techniques are completely non-invasive, as GBCAs are not injected. The magnetic labelling of arterial spins occurs upstream of the voxel of interest into which it flows, meaning that the arterial transit time (ATT) needs to be known. As with GBCA-based techniques, the similar *T*_1_ of blood/brain water renders standard ASL techniques insensitive to water exchange; the following techniques have been proposed to improve this. Multi-echo time (Multi-TE) ASL techniques allow the *T*_2_ of the labelled spins to be estimated, and infer that the *T*_2_ increase associated with increased post-label delay (PLD) time is a result of labelled water experiencing the longer *T*_2_ environment of the extravascular compartment [84, 138, 212]. This is used to calculate the pre-exchange lifetime of water, although these calculations depend on accurate ATT measurement. Tissue/blood *T*_2_ is also oxygen-dependent, complicating comparisons between studies where arterial and capillary pO_2_ may differ between groups. Diffusion-weighted (DW)-ASL exploits the difference in apparent diffusion coefficient between vascular and extravascular compartments, which can be coupled with ASL to quantify the proportion of label in each compartment as a function of PLD [205, 213]. Double-diffusion encoding methods for measuring water exchange are based on diffusion-exchange spectroscopy (DEXSY). These are known as filter-exchange imaging (FEXI). They do not rely on contrast agents or spin labelling and instead aim to harness the natural differences in water diffusion, or pseudo-diffusion, between extravascular and intravascular compartments. Intravoxel incoherent motion, originally proposed by Le Bihan et al. [137], describes the perfusion of spins in the intravascular (capillary) compartments as mimicking isotropic diffusion. The first diffusion-encoding block aims to null spins from the fast-diffusing compartment (in this case, the intravascular compartment). Spins are allowed to exchange for a given mixing time, then a second diffusion encoding block encodes spins with a second diffusion weighting. If spins exchange during the mixing time, then the measured apparent diffusion coefficient will increase with mixing time, the rate of recovery being dependent on the exchange rate [214–216]. This method has been used primarily to measure transcytolemmal water exchange but has recently been applied to study BBB water exchange [211]. This study yielded encouraging results, reporting exchange rates in the range of previously published data using other techniques. However, FEXI methods are not without limitations. They typically use a simplified ‘apparent exchange rate’ model which ignores the effects of relaxation rate differences between compartments [217], and the effects of longitudinal storage crusher gradients [218], both of which can introduce substantial biases into exchange rate estimates.

One of the major challenges with water exchange imaging is distinguishing the signals from each compartment with sufficient accuracy and precision, while ensuring the water dynamics within each compartment are accurately modelled. The extravascular compartment, for example, is often modelled as a single well-mixed compartment but in reality is composed of different cell types acting as distinct microcompartments with different NMR properties and cell membrane permeabilities [214, 219]. Validating such techniques is also complicated, due to the varied pathways water can take between compartments, especially the BBB. This multifactorial transport route means that, whilst these techniques may be more sensitive to BBB leakage than traditional GBCA-based methods, they may be less specific and more vulnerable to bias. A recent small-scale clinical study demonstrated regional heterogeneity in correlation between DCE and ASL water-exchange MRI measurements [130], suggesting that different mechanisms underlie the transport of GBCAs and water. This highlights the ambiguity in interpreting water exchange data; altered exchange rate may reflect passive paracellular diffusion, or altered flux through transporters, such as aquaporins and GLUTs [201, 220]. Alternatively, changes may be driven by altered metabolic turnover, as transcytolemmal water exchange has been reliably correlated with Na^+^/K^+^-ATPase, indicating an active contribution to water exchange [204, 221, 222].

#### MPIO and USPIO MRI

Superparamagnetic compounds, such as iron-oxide particles, induce *T*_2_ dephasing, presenting as signal voids on *T*_2_- and *T*_2_*-weighted images. A major advancement in MRI contrast agents has been the development of antibody-conjugated micro-sized particles of iron oxide (MPIO) [223]. Coupled with the molecular specificity of antibody binding, these conjugates facilitate direct, minimally invasive in vivo imaging of molecular targets present on the luminal surface of the BBB. Smaller iron-oxide contrast agents may also be used to image within the brain but these require low molecular weight to surpass the BBB, which reduces the concentration of iron delivered and precludes antibody conjugation, dramatically reducing both sensitivity and specificity [143].

Endothelial activation during inflammation stimulates the upregulation of CAMs (e.g. VCAM-1, ICAM-1) and selectins, involved in leukocyte adhesion, rolling, and diapedesis. These molecules may therefore be utilised as indicators of vascular inflammation to monitor disease progression and/or the effects of therapeutic interventions. The first study using MPIO-enhanced MRI demonstrated upregulation of VCAM-1 following intrastriatal IL-1β administration [223]. Subsequently, more sensitive contrast agents have identified cerebrovascular VCAM-1 upregulation in preclinical models of disease (AD, vascular dementia, experimental autoimmune encephalomyelitis (EAE)) and in acute systemic challenges (LPS, ethanol, hyperglycaemia), and the void volume was shown to correlate well with ex vivo measures of mRNA and protein concentration assessed using qPCR and Western blot [224]. In particular, the use of MPIOs appears promising to track the time course of inflammation for early diagnosis and image-guided therapy in chronic diseases. For example, in cases of tumour metastasis to the brain, average survival post-diagnosis is only 6 months [225]. This is largely due to late detection using existing methods (gadolinium-enhanced MRI). In three xenograft models of micro-metastatic human tumours (breast carcinoma, lung adenocarcinoma, and melanoma) in mice, MPIO MRI detected cerebrovascular VCAM-1 upregulation several days prior to detection of micro-metastases using gadolinium-enhanced MRI [226]. This identifies VCAM-1 as a potential biomarker for disease progression and indicates the potential for the improved diagnostic potential of VCAM-MPIO MRI relative to the existing gold standard. However, the authors highlight that VCAM-1 upregulation was detected up to 150 µm from the nearest micro-metastases in the histological examination, reducing the applicability of VCAM-MPIO MRI to precision image-guided treatments. P-selectin-MPIO MRI has also shown diagnostic promise; spinal cord imaging of P-selectin expression predicted both relapse and remission in a murine EAE model, suggesting benefits in monitoring patients with relapsing–remitting multiple sclerosis [148]. The P-selectin expression has also been used to image vascular inflammation in response to transient ischaemic attack [227], an event which often precedes stroke in humans but is difficult to diagnose. Importantly, the authors were able to distinguish transient ischemia from models of migraine and epilepsy, which showed no significant increase in void volume relative to control. It should be noted that these were side-by-side comparisons. This discriminative capacity may be reduced in individual animals or patients, particularly given that P-selectin is upregulated in numerous diseases which may display similar patterns of upregulation. If distinct disease-specific patterns of upregulation can be characterised, then the diagnostic potential of MPIO MRI will be improved. Numerous other preclinical studies have utilised MPIO MRI to image cerebrovascular inflammation in a range of preclinical disease models [151, 152, 154, 155, 228]. Whilst these have focused on CAM and selectin expression, countless antibodies are available to target proteins expressed in the luminal endothelium, suggesting that the true versatility of MPIO MRI has yet to be explored. However, antibody binding is likely to affect the function of target proteins. Consequently, MPIO MRI may not be viable for essential transport proteins such as GLUT1 if a high enough concentration of antibody was injected to block transport function. They may still prove useful in studying alternative inflammatory mediators and luminal BBB components in vivo.

Key features for ideal MPIO contrast agents to image the brain endothelium have been identified by Gauberti et al. [143]: the binding affinity of the antibody should be high to withstand the shear force of blood flow and the biological half-life of the conjugate in blood should be short to enable washout before imaging. The contrast agent should also be large enough to prevent false positive measurements caused by extravasation of the contrast agent to the brain parenchyma, where it would be protected from clearance mechanisms. The size of MPIOs promotes phagocytic clearance from the blood via the reticuloendothelial system, resulting in a very short blood half-life in the order of seconds to minutes (despite accumulation in organs such as the liver and spleen) [150, 153]. Conjugation to antibodies also prevents extravasation unless disruption is very severe, thus binding affinity is the major factor here and careful antibody selection is paramount.

The heavily *T*_2_*-weighted sequences used to image MPIOs are vulnerable to artefacts, such as dephasing associated with the BOLD effect, as reviewed by Gauberti et al. [143]. This obscures the MPIO signal in diseases such as stroke and tumours, in which CAMs play an important role and in which there are profound changes in blood oxygenation in and around the lesions. The same group overcame these false positive effects by pre-treating mice with oxygen to normalise haemoglobin oxygenation across the lesion prior to imaging, yielding impressive molecular images of VCAM-1 in a murine stroke model [229]. The authors also assessed behavioural metrics and showed no effect of this treatment on the mice. However, no experiments were performed to assess whether the oxygen treatment itself affected the expression of VCAM-1; the possibility that the intervention may alter VCAM-1 expression should be investigated further by analysing VCAM-1 protein and mRNA expression ex vivo with and without oxygen treatment. The dramatic effect of oxygen treatment also highlights the importance of carefully controlled administration of anaesthetic carrier gas in such studies, particularly if anaesthesia is prolonged.

The size of iron-oxide particles has profound effects on their applications for imaging. Ultra-small particles of iron-oxide (USPIOs; < 50 nm) are smaller than MPIOs (in the micrometre range), which affects their blood half-life, *T*_2_-dephasing, and interactions with/diffusion through the BBB. The smaller particles are less susceptible to phagocytic clearance via the reticuloendothelial system, which contributes to a much longer half-life than MPIOs, in the order of several hours [150]. The reduced size also confers reduced density of dephasing particles, hence the expression of an equal number of molecular targets would present as a much weaker signal void in USPIO MRI compared with MPIO MRI [150]. Computer simulations also suggest that these smaller particles would interact less with the endothelium, suggesting there may also be less antibody-target interaction, exacerbating the sensitivity deficit [230]. USPIOs have, however, proved useful in tracking the movement of leukocytes across the BBB. Leukocytes can be loaded with USPIOs ex vivo and reintroduced to the vasculature. In this instance, the resistance to degradation is beneficial, as it allows sufficient time for leukocytes to circulate to sites of inflammation and enter the CNS. Whilst circulating leukocytes can be labelled in vivo by taking up intravenously administered USPIOs, this limits specificity due to passive diffusion through the BBB. USPIOs can also be conjugated to antibodies in the same manner as MPIOs, and despite reduced sensitivity, in the absence of biodegradable coatings for MPIOs, USPIOs have a favourable safety profile. Accordingly, there are FDA-approved USPIO agents available, such as ferumoxytol and ferumoxtran-10, which have been used to image CNS tumours, neoplasms, MS, stroke, and inflammation, as reviewed by Gkagkanasiou and colleagues [149].

The main barrier to clinical translation is the use of non-biodegradable sheathes to encapsulate MPIOs; as of yet, there has been little success in generating agents which combine the biodegradability of USPIOs with the sensitivity of MPIOs. Iron administration is well tolerated clinically at higher levels than those used in preclinical MPIO studies [143]. The capsules, however, prevent degradation, resulting in bioaccumulation in the reticuloendothelial system [146]. Biodegradable alternatives would allow for the breakdown of the contrast agent and subsequent recycling of iron by the body. Perez-Balderas et al. achieved this using MPIOs encapsulated by an amine-functionalized dextran coat and demonstrated its ability to image VCAM expression following intrastriatal IL-1β administration in mice [156]. However, the sensitivity is lower than that of other agents and was tested in a severe model of acute inflammation. In order to assess whether their contrast agent may improve the early detection of pathology, it should be validated longitudinally in models of chronic disease. Other factors to consider relate to the development of sequences that retain high sensitivity at clinical field strengths; due to partial volume effects, the signal intensity from MPIOs is proportional to spatial resolution, as discussed by Gauberti et al*.* [146]. A novel type of tracer has recently been developed using dopamine-coated magnetite nanocrystals, which self-assemble to form microsized matrix-based magnetic particles (M3P) [231] in an attempt to develop a fully biodegradable tracer that retains high sensitivity. Biodegradability was assessed in macrophage culture, where the M3P particles were fragmented but commercial MPIO particles remained intact. This was supported by in vivo MRI imaging of the visceral organs of mice. Signal voids indicating accumulation in the liver were observed up to 24 h post-MP3 injection, and in the spleen up to 7 days post-injection, but these returned to baseline at later time points, suggesting degradation or excretion of the superparamagnetic particles. When conjugated to an antibody, M3P clearly detected VCAM-1 upregulation following intrastriatal LPS administration, with voids increasing at higher doses of LPS. M3P also elicited larger voids in a direct comparison with USPIO MRI, highlighting its high sensitivity, and VCAM-1 upregulation was also observed following ischemic stroke induction, a more clinically relevant model of inflammation. M3P, therefore, is a highly promising alternative to MPIO or USPIO tracers which may be fundamental in enabling the clinical implementation of highly sensitive targeted MRI contrast agents.

#### Glucose CEST/CESL MRI

**Fig. 6:**
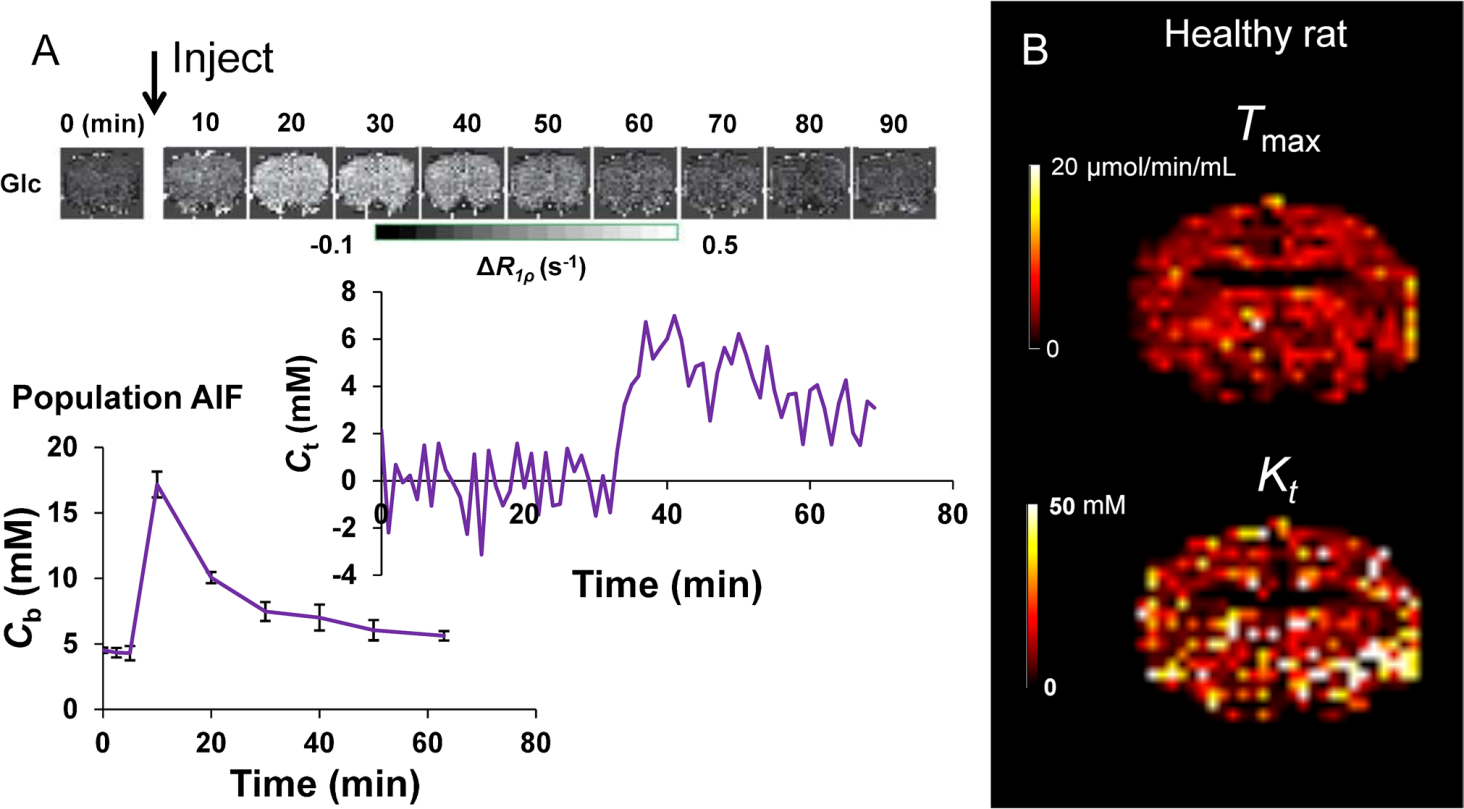
Dynamic glucoCESL MRI for the estimation of glucose transport across the BBB. **A** D-glucose is injected during the dynamic collection of *R*_1ρ_-weighted images. This enhances the signal, first in the blood, and later in the tissue as the glucose is transported across the BBB. At each tissue voxel, the change in glucose concentration from baseline (Δ*C*_t_) over time is calculated from the change in *R*_1ρ_ using known *R*_1ρ_ relaxivity of glucose, usually measured before the experiment in a test object. A kinetic model can be fitted to this data in order to calculate the maximal rate of transport (*T*_max_) and the half-saturation constant of the glucose transporters (*K*_t_) in each voxel of the brain [240]. **B** Example *T*_max_ and *K*_t_ images in a Sprague–Dawley rat (unpublished data)

Unlike water, which can take varied routes through the BBB, certain molecules required by the brain for specific purposes, such as glucose and amino acids, are transported across cell membranes via specialised transport proteins. For example, glucose transport is tightly regulated via GLUTs on endothelial cells, astrocytic endfeet, and neurones [201, 232–234]. Disturbances to this system are well-documented in neurodegenerative diseases and typically accompany metabolic dysfunction [72, 235]. Recently, glucose-sensitive MRI techniques—glucose-enhanced chemical exchange saturation transfer (glucoCEST), and glucose-enhanced chemical exchange spin lock (glucoCESL)—have been developed, which can probe glucose transport and metabolism. These approaches appear capable of quantifying glucose uptake into the brain [174, 236], and may be useful tools to probe BBB GLUT alterations in vivo. Both require intravenous injection of glucose in solution (~ 1 g/kg). GlucoCEST uses an off-resonant saturation pulse to saturate spins in glucose hydroxyl groups and encodes the signal as saturated protons from glucose exchange with unsaturated protons in water. GlucoCESL uses an on-resonant pulse to saturate water, then records the relaxation of water in the rotating frame (*R*_1ρ_), as unsaturated protons in glucose, and other labile protons, exchange with saturated protons in water. This sensitivity to labile protons from numerous molecules reduces the specificity of the technique, although by injecting a bolus of glucose and quantifying the signal change from baseline, this limitation can be largely circumvented. These techniques have been used to detect increased uptake of glucose in rodent tumour models [175, 237], and reduced uptake in rodent models of AD [236, 238, 239] with sub-millimetre resolution. However, validation of uptake against changes to GLUTs or vascular pathology has not been done. Kinetic modelling has recently been applied to this type of data to extract transport and metabolic parameters [240]. Assuming the cerebral metabolic rate of glucose is constant and saturable glucose kinetics, the rate of change of glucose in a voxel, *C* [mM], can be modelled as [240, 241]:

3 $$\dfrac{\mathrm{d}{C}(\mathrm{t})}{\mathrm{dt}}=\dfrac{{{T}}_{{max}}{{C}}_{\mathrm{b}}(\mathrm{t})}{{\mathrm{K}}_{\mathrm{t}}+{{C}}_{\mathrm{b}}(\mathrm{t})}-\dfrac{{{T}}_{{max}}{{C}}_{\mathrm{e}}(\mathrm{t})}{{{K}}_{\mathrm{t}}+{{C}}_{\mathrm{e}}(\mathrm{t})}-{\mathrm{CMR}}_{\mathrm{glc}}$$

where ${\mathrm{C}}_{\mathrm{b}}(\mathrm{t})$ [mM] is the glucose concentration in whole blood, ${\mathrm{C}}_{\mathrm{e}}(\mathrm{t})$ [mM] is the glucose concentration in the parenchyma, ${{T}}_{{max}}$ [μmol/min/mL] is the maximal rate of transport, ${{K}}_{{t}}$ [mM] is the half saturation constant of glucose transport, and *CMR*_glc_ [μmol/min/mL] is the cerebral metabolic rate of glucose utilisation. Kinetic analysis of this type may enable estimation of transport and metabolic rates, providing information on the density of glucose transporters and the relative number of each type (Fig. 6). However, it is currently challenging to obtain reliable image-derived input functions (i.e. estimates of *C*_b_(t)), which is particularly important due to individual differences in insulin responses [242]. Therefore, new approaches that provide more robust detection of image-based input functions are needed if kinetic analyses of glucoCEST and/or glucoCESL data are to be useful as research and clinical markers of glucose uptake and metabolism.

Substantial validatory work has been performed comparing glucoCEST and CESL uptake to that of other glucose analogues or tracers such as non-metabolisable 3OMGc [243], partially metabolised 2DG [173], and intravascular agents L-glucose [175] and mannitol [174], and across different conditions such as altered anaesthesia and dose [173, 174]. However, validation against transport and TJ protein levels at the BBB, glial activation or hexokinase activity has not been done.

Chemical exchange MRI is an exciting area of development, as it could potentially be applied to any molecule with labile protons exchanging in the detectable range, thereby facilitating non-invasive studies into BBB transport and metabolism of amino acids, for example. These developments may help develop a more comprehensive understanding of disease-specific BBB pathology. The techniques are limited in that they require a large (in the order of 1 g/kg) injection of glucose, which may itself alter the distribution of transporters or osmotically increase the paracellular permeability at the BBB. A recent study demonstrated that xylose can be infused as a more sensitive (hence lower dose) agent than glucose in both CEST and CESL [244]. 2-Deoxy-glucose (2DG), a non-metabolised analogue of glucose, has also been used as a more sensitive agent [238] but is toxic at the doses required for detection.

### Nuclear imaging techniques

**Fig. 7:**
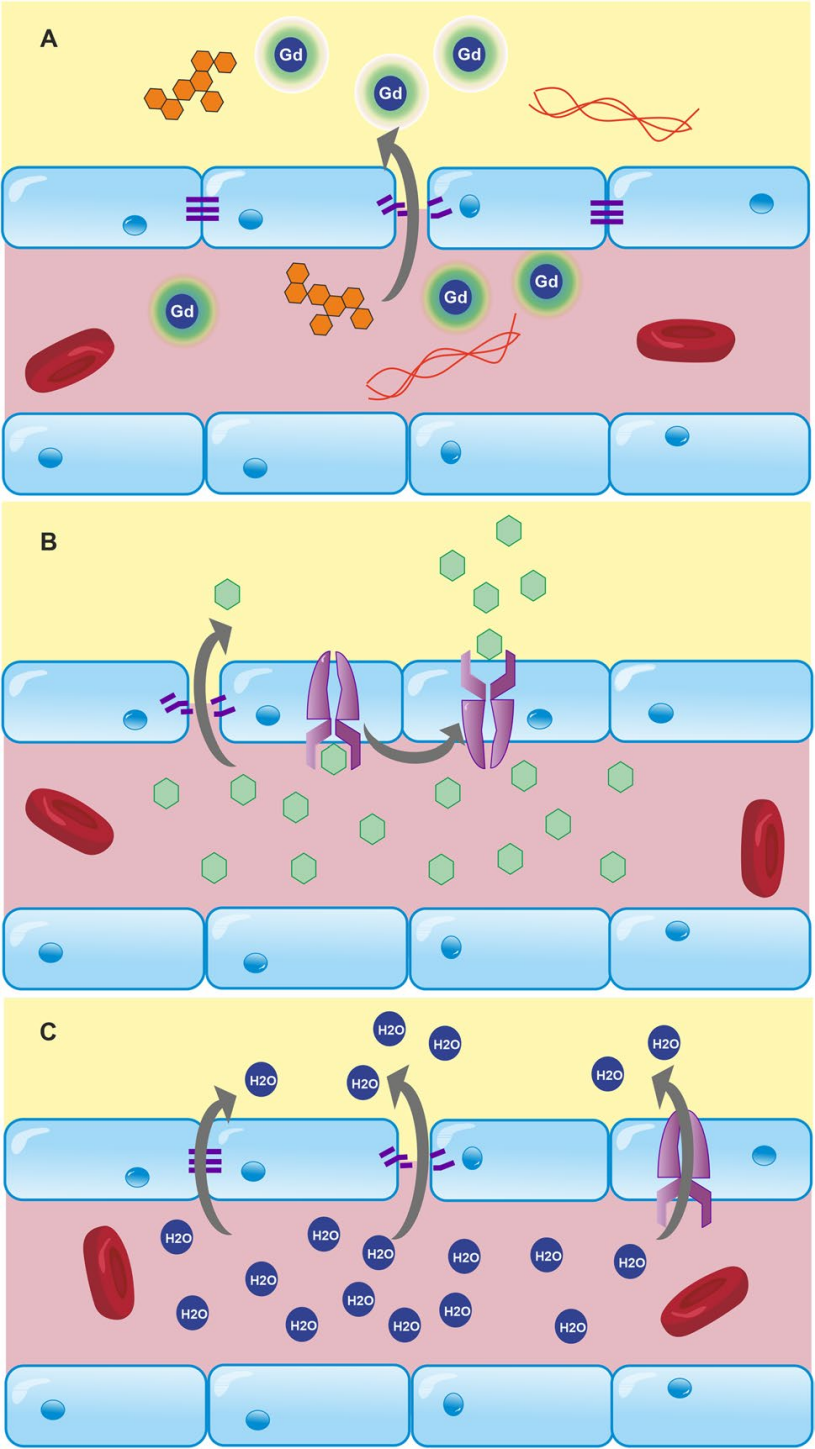
Different markers indicate different aspects of BBB function/dysfunction. **A** Tight junctions restrict the majority of molecules from crossing the BBB. Detection of molecules such as dextrans and fibrinogen is generally considered to indicate that these junctions are impaired, as these molecules do not have specific transport mechanisms. **B** Molecules such as glucose and amino acids have specific transport proteins, so alterations in the transport of these substances may indicate disruption to these mechanisms. Alternatively, they may indicate changes to free diffusion resulting from tight junction impairment. **C** Very small molecules, such as water, are able to cross the BBB even when junctions are intact, although the rate of exchange increases when junction integrity is compromised. Water is also a cofactor for many transport proteins, including GLUT1. These numerous routes of movement across the BBB complicate the interpretation of readouts from water-exchange techniques

MRI is a powerful technique with moderate to high resolution and structural and functional imaging capacity. Coupled with the availability of endogenous or easily administered tracers, it is immensely useful in BBB studies (Fig. 7). However, the poor sensitivity and specificity of MRI limit its use for molecular imaging notably of active transporters. Positron emission tomography (PET) and single-photon emission computed tomography (SPECT) are nuclear imaging techniques, which sacrifice some spatial resolution but have exquisite sensitivity and are considered gold standard techniques for in vivo imaging of transport mechanisms such as P-gp-mediated efflux, and GLUT1-mediated glucose uptake from the blood.

Positron emission tomography (PET) is an ionising nuclear imaging technique sensitive to positrons, which are released from radiotracers via beta decay. These positrons travel short distances before interacting with their antiparticles, electrons, in annihilation events which produce two antiparallel photons per event. These photons travel as gamma rays in opposite directions, which facilitates coincidence detection by scintillation counters for reconstruction into images. In contrast, SPECT radiotracers directly emit single photons. This yields fewer photons than PET, and these events cannot be localised via coincidence detection, which reduces spatial resolution relative to PET.

#### Imaging efflux

**Fig. 8:**
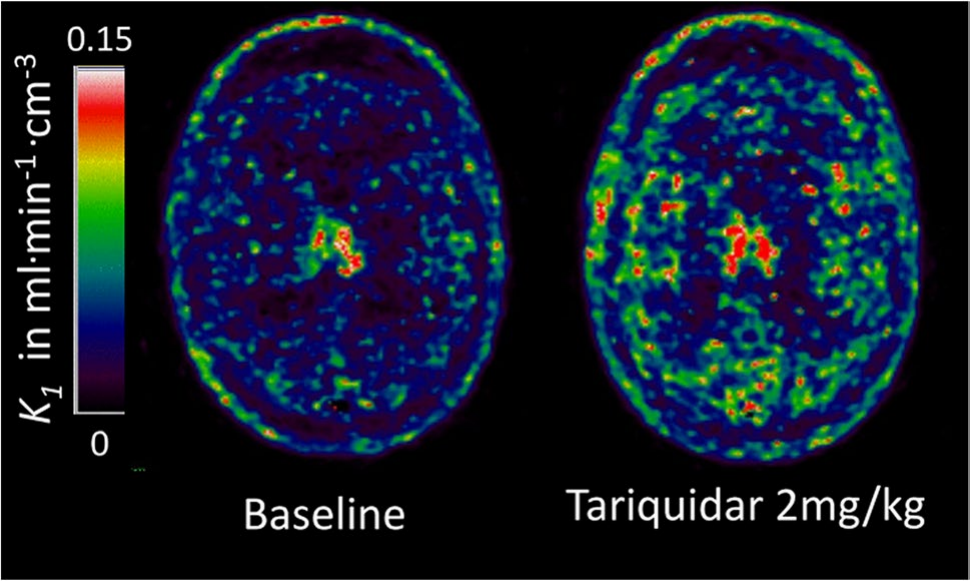
Parametric maps of the plasma-to-brain transport rate constant *K*_1_ of the P-glycoprotein (P-gp) substrate PET tracer (R)-[^11^C]verapamil estimated from the single-tissue compartment model with blood volume using a metabolites-corrected plasma input function. Administration of the P-gp inhibitor Tariquidar at 2 mg/kg increased brain uptake by 54% compared to baseline in this 36-year-old healthy male volunteer. The images kindly provided by Dr Marie-Claude Asselin were acquired as part of the EURIPIDES study [267]

Efflux transporters are densely expressed at the BBB and are fundamental to the regulation of homeostasis within the CNS (Fig. 8). These predominantly belong to the ATP-Binding Cassette (ABC) family and include the transporters P-gp, BRCP, and MRP, each of which can actively transport a variety of compounds from the CNS to blood and there is a large degree of redundancy between them [245–247]. These systems are known to falter in many diseases [248] and they also present a major barrier to CNS drug delivery, contributing to multidrug resistance in epilepsy and cancer, for example [249, 250]. There is also a subtler decrease in efflux function at the BBB in normal ageing, a factor that has been identified as a potential cause of worsened drug side-effects in elderly patients [251].

**Fig. 9:**
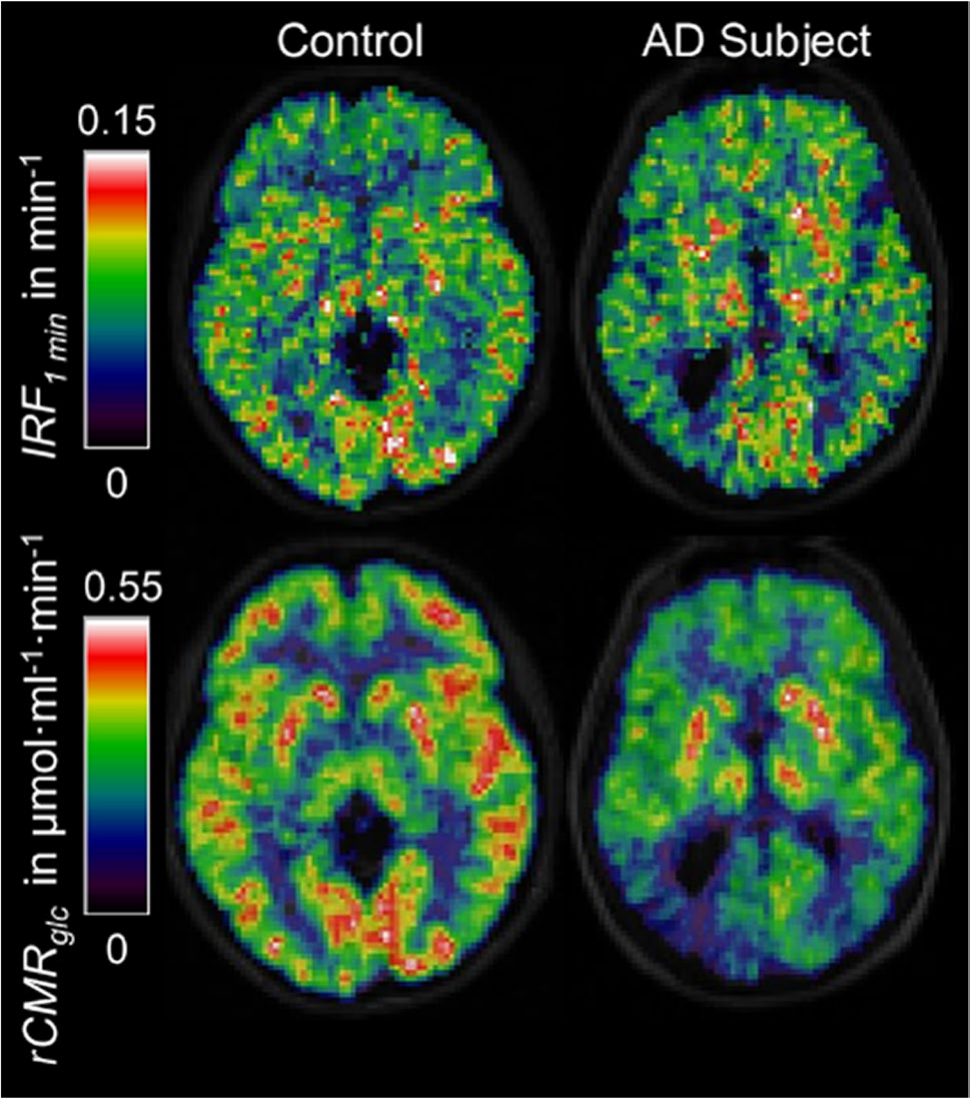
Parametric maps from [^18^F]fluorodeoxyglucose brain PET scans in an AD subject (right column) and a control participant (left column) calculated with spectral analysis [285]. The top row shows the values of the impulse response function at 1 min which are similar (although not identical) to the plasma-to-brain transport rate constant *K*_1_. In the bottom row, regional cerebral metabolic rates of glucose are shown with clearly lower rates in the cortical grey matter of the AD subject than the control participant. As a characteristic imaging feature, the glucose hypometabolism is particularly noticeable in the parietal cortex of the AD subject with marked left–right asymmetry. In contrast to rCMRglc, the differences between the two IRF1min images are much smaller. Images reproduced by courtesy of Edison et al. [286]

[^11^C]-verapamil and its enantiomers [252] have been used to quantify the function of the efflux transporter P-gp (Fig. 9), three of which ([^11^C]-verapamil, [^11^C]-N-desmthyl-loperamide and [^11^C]-metoclopramide) have been approved for clinical use [178]. Three types of radiotracers have been developed: efflux transporter substrates, inhibitors, and pro-drugs. Radiolabelled substrates are by far the most studied and are the major focus here. They have been used to demonstrate that P-gp over-expression confers multi-drug resistance in treatment-refractory tumours [253] and epileptic foci [254, 255] by blocking the access of drugs to their targets. Conversely, degenerative diseases like AD [177, 256] and PD [257] are linked to the downregulation or loss of function of these transporters, which impairs the clearance of amyloid and other neurotoxic compounds, potentially driving or exacerbating neurodegeneration by further disrupting homeostasis. The use of modulators, such as the P-gp inhibitor cyclosporin A (CsA), has helped validate measures of P-gp function. For example, the volume of distribution of [^11^C]-Verapamil increases dose-dependently with increased CsA [258]. Verapamil is considered the gold-standard tracer to assess P-gp function, primarily because of its high selectivity for P-gp at nanomolar concentrations [259], but also due to its good reproducibility and ability to detect subtle changes, such as those which occur in normal human ageing [260, 261]. However, the lipophilicity and metabolite profiles of verapamil are below optimal, as reviewed by Luurtsema et al. [176]. Verapamil (along with other radiolabelled P-gp substrates) has high affinity for P-gp and therefore low initial standardised uptake values; this limits their potential in studying differences in transporter function (extrusion) between brain regions without P-gp blocking with CsA or tariquidar [262]. Furthermore, partial volume effects can make it difficult to study small brain regions such as the hippocampus, due to the higher signal in the nearby choroid plexus[263], which can be a limitation in diseases with a significant hippocampal component, such as AD or epilepsy. One strategy to develop improved P-gp tracers is to identify substrates or inhibitors with less affinity to the transporter (e.g. [^11^C]-Metoclopramide and [^18^F]-MC255), which allows for higher initial brain uptake and, therefore, greater capacity to assess P-gp function [176]. In rats, [^18^F]-MC255 demonstrates a high volume of distribution and metabolic stability and is not affected by BCRP inhibition, suggesting it has good selectivity for P-gp [179]. [^11^C]-Metoclopramide has also shown promise in rodents and non-human primates and humans, with similar selectivity for P-gp [264–266]. Tracers have been developed to study the other major efflux transporter families (breast cancer resistance proteins and multi-drug resistance proteins), although there is poor specificity between families.

#### Imaging paracellular integrity

Whilst DCE-MRI remains the most common method of imaging BBB integrity in vivo, a number of radiotracers have been investigated in attempts to utilise the higher sensitivity of PET to improve detection.

The amino acid 2-aminoisobutyric acid (AIB) is restricted from the brain by the healthy BBB, has a molecular weight of 103 Da, is metabolically stable, and can be readily labelled with ^11^C, making it a viable candidate to assess BBB permeability [268]. [3-^11^C]-AIB has demonstrated promise in cancer diagnosis, comparing preferably to FDG-PET in discriminating between tumours and normal tissue with regard to BBB impairment and hypermetabolism, respectively [140]. Subsequently, a more detailed validatory study investigated the tracer in two models of BBB opening: focused ultrasound and LPS in young rats [141]. The study also made limited comparisons with DCE-MRI. Several advantageous characteristics were identified here: firstly, the tracer kinetics in plasma and whole blood were not found to differ, suggesting that aortic image-derived arterial input functions may be possible, reducing the technical skill required for studies and minimising discomfort for patients in the event of clinical translation. Secondly, enhanced unidirectional blood–brain transfer constant (*K*i) was detected in both models relative to the contralateral hemisphere; this decreased over the course of 60 min following sonication—as expected in the acute model of BBB opening—but remained significant for the duration of the session. Furthermore, analysis by autoradiography and Evans Blue microscopy showed a strong correlation between in vivo imaging and high-resolution ex vivo methods. Finally, the SNR of PET imaging increased during the 60-min imaging session, whereas DCE-MRI SNR peaked at around 10 min. No comparisons were made between the sensitivity of these two techniques, however, which would have been a valuable comparison.

Direct comparisons with DCE-MRI will be essential in evaluating the efficacy of these radiotracers. This is highlighted by Breuer et al., who compared PET ([^68^ Ga]DTPA), SPECT ([^99m^Tc]DTPA), and DCE-MRI (Gd-DTPA) in a pilocarpine model of epileptogenesis in female rats [269]. All techniques detected BBB impairment in the model, predominantly in the hippocampus. Overall, DCE MRI outperformed both nuclear techniques in terms of sensitivity which could be due to various factors. First, the relationship between the MR signal and Gd concentration may not be linear whereas SPECT/PET provides an exact quantification of the tracer concentration. Second, MR Gd-contrast agents are administered in concentration in the milligram range, hence providing a very strong signal with optimised Gd-detection *T*_1_ sequence, whereas PET and SPECT tracers are administered in the nano- to microgram range, which has the advantage of limiting possible undesirable effect or accumulation due to the high concentrations used in MRI. Third, the relatively low resolution of SPECT and PET when compared with the size of the ROIs may lead to some partial volume effect (spill-over), hence leading to an under-estimation of the signal. Finally, the authors pointed out that previous studies had shown that DTPA [^68^ Ga] complexes might be less stable than with other chelators, leading to free [^68^ Ga] being released in the blood and associating with plasma protein transferrin which can be actively transported across the BBB, therefore reducing the specific [^68^ Ga]DTPA to noise ratio in the ROIs. However, the authors also noticed in the cerebellum that DCE MRI detected a BBB leakage which was not observed ex vivo with FITC-albumin. This is likely due to the difference in molecular weight between GBCAs and albumin, but may also suggest that, in some instances or some brain ROI, DCE-MRI may be affected by in situ *T*_1_ signal or that DTPA compounds and FITC-albumin are not exactly diffusing across the BBB in the same way [269]. Other tracers, such as [^18^F]2-Fluoro-2-deoxy-sorbitol, have been developed to investigate paracellular permeability notably in the focused ultrasound model [139]. Whilst the tracer appears sensitive and reproducible in this model of BBB opening by FUS, this tracer needs to be evaluated in a more clinically relevant model of disease in which more subtle BBB openings are present.

On another hand, PET imaging may also prove useful to evaluate the permeability of the BBB to nanoparticles with potential therapeutic perspectives as illustrated by Debatisse et al. [270]. However, such application is only relevant in case of severe BBB alterations, such as in stroke, due to the fairly large size of such nanoparticles (~ 10 kDa).

Overall, PET and SPECT offer much greater sensitivity than MRI techniques at the cost of resolution and exposure to radioactivity, although this improvement in sensitivity is yet to be demonstrated experimentally and will require developments and optimisation of new small molecular weight tracers. Radiolabelling techniques also provide potential access to smaller molecules than classical Gadolinium contrast agents used for DCE-MRI without the requirement of a chelator. This is of particular importance, as smaller contrast agents are required to assess subtler alterations of the BBB which may not be detected with classical Gd-based DCE-MRI contrast agents. In the case of MR measure, water diffusion through the BBB is of great interest (see ‘Water-exchange MRI techniques for probing BBB function’ section) while other, more effective, PET tracers of low molecular weight may be considered in the future.

#### Measuring glucose transport across the BBB: [^18^F]FDG-PET

[^18^F]-fluorodeoxyglucose (FDG) is the [^18^F] radiolabelled form of 2-DG, a functional substrate of GLUT1, hence it is extracted from the blood across the BBB via the same mechanisms as glucose, but does not undergo further metabolisation after phosphorylation into FDG-6P by hexokinase [271–273]. In contrast to glucose, this results in the accumulation of [^18^F]FDG in cells allowing accurate quantitative measurements of cerebral metabolic rate for glucose utilization (*CMR*_Glu_). [^18^F]FDG PET has demonstrated cerebral glucose hypometabolism in AD in a reproducible symptom-relevant pattern (Fig. 9) [274–276] with the capacity to distinguish AD from MCI and cognitively normal individuals [277, 278]. [^18^F]FDG PET has, therefore, excellent diagnostic potential and has been used extensively to stratify MCI and AD patients before β-amyloid specific tracers such as [^11^C]PIB or [^18^F]Florbetaben became available [76, 279–281]. Hypometabolism has been observed in several neurodegenerative diseases and its anatomical distribution is disease-specific. Moreover, hypometabolism indicated by FDG-PET is a good predictor of imminent cognitive decline [72, 278], unlike amyloid, which may build up in the AD brain for decades prior to clinical symptoms. However, it remains unclear whether hypometabolism is a cause or consequence of reduced neuronal activity linked to synaptic loss [282, 283]. This is further complicated by the proposed multicellular mechanism of glucose and lactate transport, known as the astrocyte-neurone lactate shuttle (ANLS) [66, 78, 232]. Furthermore, the measures are affected by CBF and the permeability surface-area product of glucose, which are both affected by ageing and disease [29, 284].

Whilst FDG-PET is typically used to quantify metabolism, the early signal readout is also linked to transport. Measuring this accurately is dependent on measuring an accurate input function, preferably through arterial blood sampling during dynamic scanning. This sampling facilitates the determination of *K*_1_, a measure of uptake across the BBB via GLUT1.

Modelling of FDG-PET kinetics is similar to that of glucoCESL/CEST, however, since only trace amounts of FDG are administered, rate constants *K*_1_ and *k*_2_ are not concentration-dependent. Furthermore, because FDG is trapped in cells as FDG-6-phosphate, the contribution from glucose metabolites is non-negligible. The rate of change of FDG in the free glucose compartment is given by [273, 287]:

4 $$\dfrac{{\mathrm{d}{C}}_{\mathrm{f}}(\mathrm{t})}{\mathrm{dt}}={{K}}_{1}{{C}}_{\mathrm{p}}(\mathrm{t})-{{k}}_{2}{{C}}_{\mathrm{f}}(\mathrm{t})-{{k}}_{3}{{C}}_{\mathrm{f}}(\mathrm{t})$$

where *K*_1_ [min^−1^] is the rate of tracer influx across the BBB, *k*_2_ [min^−1^] is the rate of tracer efflux from brain-blood, *k*_3_ is the rate of phosphorylation of FDG into FDG-6-P, *C*_p_ is the concentration of FDG in plasma, and *C*_f_ is the concentration of FDG in tissue. These parameters can be used to calculate *K*_i_:

5 $${K}_{i}= {K}_{1}\left(\dfrac{{k}_{3}}{{k}_{2}+{k}_{3}}\right)$$

The rate of change of the concentration of FDG-6-phosphate in the metabolised compartment is:

6 $$\dfrac{{\mathrm{d}{C}}_{\mathrm{m}}(\mathrm{t})}{\mathrm{dt}}={{k}}_{3}{{C}}_{\mathrm{f}}(\mathrm{t})$$

It is common to also model a rate of dephosphorylation from FDG-6-P to FDG using an additional rate constant *k*_4_ [287]. However, since FDG is largely trapped in cells upon phosphorylation and the rate of dephosphorylation is slow, *k*_4_ may be treated as negligible for the analysis of dynamic data from FDG-PET scans [288]. The total concentration of radioactivity is given by:

7 $${C}= {{{C}}_{\mathrm{f}}+{ C}}_{\mathrm{m}}$$

This two-compartment kinetic model has been used in the FUS model of BBB disruption, in which *K*_i_ of FDG was significantly lower in sonicated rat brains compared to control rats immediately after sonication [289]. Interestingly, this was true in both hemispheres, despite sonication being directed only upon the right hemisphere. This was supported by Western blot, which showed reduced global GLUT1 expression, demonstrating that FUS induces a transient downregulation of cerebral GLUT1 and that this can be detected via FDG-PET. Similar studies could be performed in preclinical disease models to assess whether FDG-PET can detect changes in glucose transport in more physiologically relevant cases.

FDG-PET is not without limitations, however. Accurate assessment of glucose uptake and metabolism using FDG-PET requires the image analyst to mathematically account for differences in transport and phosphorylation between FDG and glucose. This is done using the experimentally-derived lumped constant (LC), which is dependent on the relative expression of glucose transporters and relative contributions of transport and phosphorylation. The LC has been shown to be variable when calculated in different labs, as well as between brain regions and in lesions, particularly tumours [290]. This variability may introduce bias into imaging studies if the LC is not accurately calculated with appropriate spatial resolution. Despite these limitations, kinetic analysis of FDG uptake across the BBB appears adept at detecting changes in uptake associated with reduced GLUT1 expression, and studies directly comparing this with glucose-sensitive MRI methods will be important in understanding the relative merits of each modality.

#### Imaging of BBB molecular components

Tracers capable of investigating other aspects of BBB function are less established than verapamil and FDG, although a number are being developed. Aquaporin radiotracers, for example, have the potential to investigate water exchange. One such tracer is [^11^C]TGN-020, which is capable of distinguishing between clinical stages of astrocytoma [180]. It binds to both AQP1 and AQP4 [291], predominantly expressed in the BCSFB and BBB, respectively [292]. The poor spatial resolution of nuclear imaging, and binding to both AQP4 and AQP1, may cause quantification errors in the boundaries between these barriers where they cannot be easily spatially distinguished.

Recently, tracers for RAGE have been developed [293]. These tracers ([^18^F]RAGER and [^18^F]InRAGER) target the intra- and extracellular domains of RAGE. They have high affinity and good brain uptake, although have demonstrated binding to other targets, such as melatonin receptors, in vitro [293]. Despite this, these tracers will make useful scaffolds to develop improved tracers, unlike previous tracers which were macromolecular and unable to cross the BBB [293].

A promising example of PET tracers being developed to improve the categorisation of lesions is that of matrix metalloproteinase (MMP)-PET, which has been used to distinguish early BBB lesions (those with active leukocyte infiltration) from existing lesions in which leukocyte infiltration has ceased [294–296]. This could be used in combination with MRI techniques to confirm whether changes seen in water exchange, for example, are affected by the pathological stage of the lesions they are associated with. Alternatively, nanobodies can be used for targeted PET to image inflammatory markers in the BBB. These have been used to image VCAM-1 in atherosclerotic lesions in mice [297] and similar tracers are being developed to image ICAM-1 [298]. These tracers have been used broadly to image immune interactions peripherally, as reviewed by Lee et al. [158], although they could also be used to image the BBB. Theoretically, these could be applied to a range of molecules, similarly to MPIO MRI, as discussed above.

### Intravital microscopy

Whilst MRI and nuclear imaging techniques are powerful in providing macroscopic information with full brain coverage and can do so in a highly specific manner to probe individual mechanisms of BBB dysfunction, they lack the spatial resolution necessary to elucidate the cellular/molecular underpinning of these observations. This resolution can be attained using intravital microscopy. High-resolution fluorescent in vivo microscopy can quantify and localise cellular components, including junctional proteins and leakage of tracers across the BBB at the level of the vessel. Multiphoton microscopy is the predominant form used in BBB research and will be the focus here.

Multiphoton imaging uses near-infrared lasers to excite coincidence-detecting fluorophores in a specific plane. This limits photodamage and increases penetrance [299]. These properties are essential for in vivo imaging as they reduce tissue damage, facilitate repeated or longitudinal imaging sessions, and increase the depth of tissue that can be imaged. Furthermore, the sub-femtolitre volume of excitation is precise enough to facilitate photolytic uncaging of signalling molecules. This enables acute experimental modulation of signalling pathways in mechanistic studies in vivo.

**Fig. 10:**
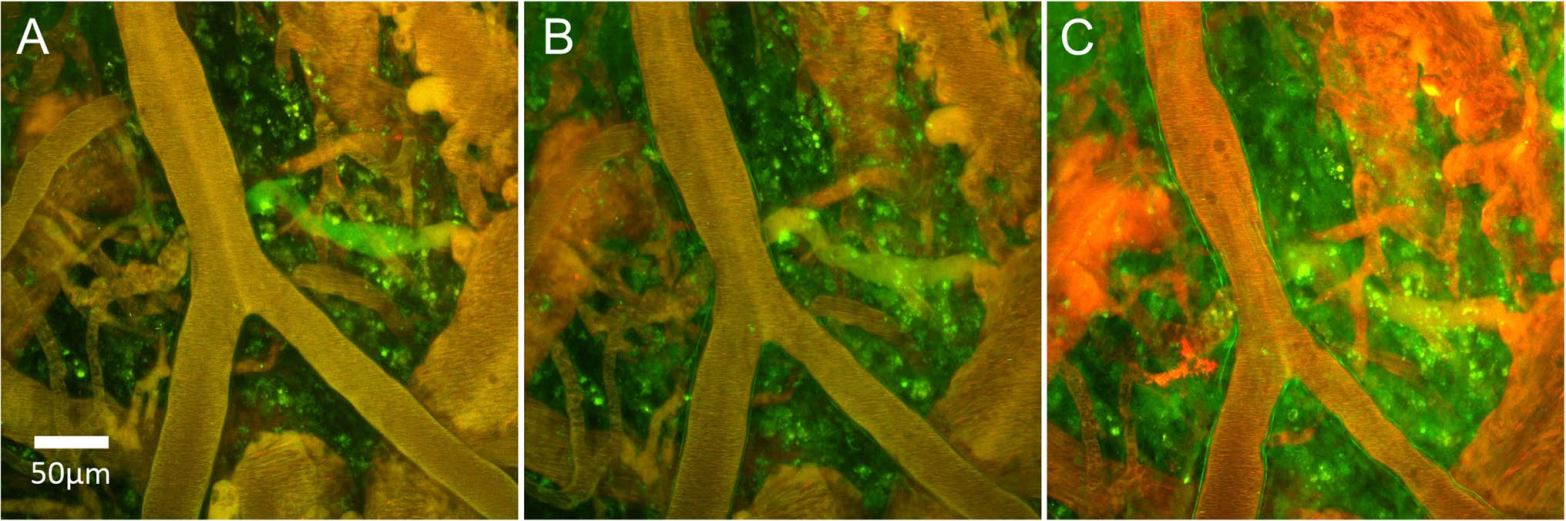
Increased BBB permeability imaged with intravital two-photon microscopy following acute experimental stroke. Changes in BBB permeability after experimental stroke: Mice were injected via tail vein with fluorescent dextran 3 kDa (green) and albumin (red). Before the procedure (**A**) and 45 min after acute middle cerebral artery occlusion (**B**), both the dextran and albumin are detected mainly in the vessel resulting in yellow appearance. In the following 2 h, there is an increase in the BBB permeability with the green dextran leaking into parenchyma while the red albumin stays inside the vessel (**C**). In vivo imaging using two-photon microscopy, Schiessl lab. Unpublished data

With regard to imaging the BBB and vasculature, two approaches are typically used: injection of dyes/leakage agents, or imaging of cell-specific fluorescent markers. Dyes, commonly fluorescent dextrans, can be used to image vasculature. Larger dextrans are retained within vessels and are useful for imaging vascular density and morphology. Smaller dextrans (< 3 kDa) can pass through the impaired BBB; this can be imaged to quantify BBB leakage (Fig. 10) [142, 300]. The applications of specific cellular/subcellular markers are diverse. For example, imaging BBB calcium signalling in NG2-creERT2;GCaMP6f mice following synaptic activation demonstrates a precise temporal pattern of the smooth muscle cell and pericyte activation, which propagates from the site of activation upstream to the pial arteriole, in order to modulate functional hyperaemia [196]. The relative contributions of mural cells to functional hyperaemia is highly contested [50] and two-photon imaging has been instrumental in distinguishing the roles of vascular smooth muscle cells and pericytes [301]. This cell type- and phenotype-specific discrimination has the potential to improve statistical power through experimental resolution (i.e. by specifically analysing one cell type or phenotype, rather than heterogeneous populations). This will be paramount in studies probing subtle dysfunction in the early stages of neurodegeneration, for example, where effect sizes are small and may be diluted by the inclusion of cells that are not involved in pathogenesis. The technique has also been used to demonstrate the accumulation of liposomes associated with increased transcellular and paracellular permeability following stroke [302]. This is proposed as a potential route for therapeutic intervention in the disease. This highlights the value of high spatial resolution in determining specific mechanisms of BBB dysfunction (i.e. accumulation of liposomes via upregulated caveolae or by disassembly of TJ complexes).

In vivo two-photon imaging has also been paramount in understanding leukocyte diapedesis [303] (Table 2). In the presence of intravascular dyes, leukocytes appear dark and can be distinguished from erythrocytes based on velocity. They can also be directly visualised via DNA-intercalating dyes [304]. Leukocyte cell type resolution can be attained by isolating the cells, purifying and labelling them in vitro, and then reintroducing them [303]. Similarly, two-photon imaging has shed light on the heterogeneous behaviour of astrocytes in glial scarring following brain injury [167, 305]. These examples highlight how intravital microscopy can be used to probe the microscopic detail underlying macroscopic changes measured by MRI and nuclear imaging. However, intravital microscopy is low throughput and highly invasive, necessitating either craniotomy or cranial thinning for optical imaging of the brain. Cranial thinning is less invasive and is necessary in older animals (it is common for the dura to attach to the skull. This can cause immediate damage and subsequent inflammation when the skull is removed from older animals). It is also well-suited to mice, whose meninges are more translucent and cranium less dense than those of rats [306]. However, this technique diminishes resolution as a result of optical scattering by the remaining cranium. The cranial window technique exerts a temporary (2–3 days) cooling effect on the brain; the use of water-immersion objectives reinstates this temperature drop and should be avoided or corrected for [307]. Craniotomy is also associated with reactive gliosis, inflammation, and oedema, which need to be minimised by careful aseptic technique and surgery [306, 308].

**Table 2:** In vivo imaging techniques

	MRI	PET/SPECT	Intravital two-photon microscopy
Field of view	Whole brain 3D	Whole brain 3D	• Typically ~ 500 µm^2^, dependent on magnification • Poor tissue penetration (600 µm)
Tissue Contrast	Good	Poor	Excellent
Ionising radiation?	No	Yes	No
Maximum spatial resolution	100 µm isotropic	• 0.83 mm and 2.36 mm for preclinical and clinical PET scanners respectively • down to < 1 mm and 8 mm for preclinical and clinical SPECT scanners respectively	< 1 µm
Sensitivity	0.1—1 mM	10 – 100 pM	100 – 1000 nM
Invasive?	No (or minimally)	No (or minimally)	Highly
Other considerations	• Vulnerable to image acquisition artefacts including Gibbs ringing, susceptibility artefacts, and spatial distortions	• Logistical issues regarding tracer synthesis	• Photodamage to tissue – although less than in other microscopy techniques • Unsuitable for clinical use

The fundamental limitations of optical imaging are difficult to overcome in vivo. For example, the optical path is scattered significantly and penetrance is limited to ~ 600 µm [46], although this can be extended slightly by using a system with excitation and emission shifted to lower wavelengths. The resolution within this visible depth is variable, as scattering disrupts the homogeneity of the excitation and emission light paths [301] and this is also variable between animals, introducing potential quantification error [306]. This penetration limit constrains our visualisation to the outer cortex. This tissue lies immediately beneath the site of craniotomy and is thus most affected by the procedure, which means any vessels imaged will be exposed to inflammation and the cooling effects associated with the cranial window. It also prevents investigation of BBB changes in deep areas, such as the hippocampus, which is believed to have neurovascular impairment in AD [24], a murine model of epilepsy [309], and rodent models of essential hypertension [310]. The penetration limit can be circumvented using two-photon endoscopy [311], although this will initiate penetration-induced inflammation [312, 313].

Furthermore, multiphoton microscopy is dependent on the availability of good markers. Some fluorophores are better suited to single-photon excitation [314] and markers may lack specificity. For example, only recently have dyes been developed that are capable of distinguishing between mural cells [315]. A potential area for future development is to produce pH-sensitive dyes capable of detecting extravasation. Higher CO_2_ concentration in tissues reduces pH relative to blood [316]; a dye that was excited in this acidic pH but not in the relatively alkaline environment of the vascular lumen would aid improve the distinction of intra-/extravascular dye and reduce variability in how this is determined between groups.

The tiny field of view (FOV) using this technique relative to MR and nuclear techniques, and the spatial restriction imposed by the cranial window, means that appropriate localisation of the craniotomy/cranial thinning is essential. This can be guided by the tomographic and MR techniques discussed above. For more precise localisation, techniques such as intrinsic signal optical imaging can be used to identify specific vessels to investigate based on changes in the oxygenation state of blood [48].

## Discussion and summary

This review discusses the fundamental strengths and limitations of in vivo imaging techniques available to study cerebrovasculature and BBB. It is clear that no one method can fully characterise the complexity of the BBB (Fig. 7), and that numerous modalities and approaches need to be combined for complete characterisation. Macroscopic imaging techniques, such as MRI and PET, can be used to identify key areas of interest and perform longitudinal studies both clinically and in laboratory animals. More invasive in vivo techniques such as intravital two-photon imaging are restricted to preclinical research but can provide high-resolution data to validate macroscopic techniques and elucidate the mechanisms by which macroscopic changes arise.

DCE-MRI has been established as the standard non-invasive method of assessing paracellular BBB integrity. The availability of small molecular weight contrast agents means they can be used to detect relatively subtle changes in permeability. Questions remain as to whether these contrast agents pass purely via junctional gaps, or whether they can also pass via transcytosis. This may be an important consideration for ischemic stroke and AD, where Cav1, a membrane protein essential for transcytosis is upregulated [302, 317]. Despite these uncertainties, DCE-MRI remains a valuable technique and numerous kinetic modelling approaches have been developed to probe the transport of contrast agents across the BBB. However, the need to detect even earlier, subtler BBB pathologies to diagnose degenerative diseases has driven the development of alternative tracers, such as water, which is both endogenous and smaller in size than GBCAs. Alternatively, the development of improved tracers to exploit the higher sensitivity of nuclear imaging techniques may provide a different route to assess the subtlest impairments to BBB integrity.

Additionally, MRI may be used to probe specific carrier-mediated transport and inflammatory mediators via glucoCEST/CESL and USPIO/MPIO imaging. These techniques have been applied in pathologies with major dysfunction; for example, glucoCESL has been applied in cancer, a disease with profound upregulation of transport and metabolic processes. To demonstrate the true potential of the techniques, they need to be shown to detect changes in a wider range of disorders with less pronounced symptoms. The deficit in glucose transport/metabolism in AD, for example, is an order of magnitude smaller than that in cancer. Combining glucoCESL MRI with intravital two-photon imaging or ex vivo analysis could provide high-resolution molecular detail to clarify changes in cortical glucose uptake, based on BBB protein expression and localisation. These data could additionally be used to assess changes to transport/metabolic apparatus which may be used to support the validation and development of kinetic models to describe glucose-sensitive MRI data. This will build confidence in the interpretation of clinical data. Characterising the biology underlying the changes in these novel MRI techniques is essential, due to the number of factors that may affect readout—both regulated transport systems and alterations in paracellular integrity may influence signal in glucose-sensitive MRI, and numerous cell types may be involved. Furthermore, direct comparisons with FDG-PET for kinetic analysis of glucose/FDG uptake will be fundamental in assessing the relative merits of each technique. The use of antibody conjugates and nanobodies for MRI and nuclear imaging is a highly promising area of development. The specificity conferred by antibodies may allow for a more comprehensive analysis of the expression of proteins in the BBB across the entire brain, although these techniques have so far only been applied to a limited range of molecular targets.

In vivo microscopy has been particularly useful in elucidating changes at the cellular level, e.g. processes involved in the modulation of haemodynamics, leukocyte migration, and diapedesis, as well as rapid cellular processes such as calcium signalling dynamics. Furthermore, the ability to image subcellular detail is valuable in characterising alterations in the thickness of the basement membrane and glycocalyx. It can also be used to assess leakage of perfused markers, which is useful in supporting readouts from DCE-MRI. However, the small FOV and technical/invasive procedures required to set up the microscope reduce the throughput of the technique.

## Data Availability

The datasets used and/or analysed during the studies described in this manuscript may be available from the corresponding author of the referenced original publications [186, 267, 286].

## Abbreviations

AD: Alzheimer’s disease
ADC: Apparent diffusion coefficient
AIF: Arterial input function
AJ: Adherens junction
ANLS: Astrocyte neurone lactate shuttle
APOE: Apolipoprotein E
AQP: Aquaporin
ASL: Arterial spin-labelling
ATT: Arterial transit time
BBB: Blood-brain barrier
BCSFB: Blood-CSF barrier
BEC: Brain endothelial cell
BK: Big current potassium channel
CA: Contrast agent
CAM: Cellular adhesion molecule
CBF: Cerebral blood flow
CESL MRI: Chemical exchange spin-lock MRI
CEST MRI: Chemical exchange saturation transfer MRI
CNS: Central nervous system
COX: Cyclooxygenase
CSF: Cerebrospinal fluid
CT: Computerised tomography
DCE: Dynamic contrast-enhanced MRI
DEXSY: Diffusion-exchange spectroscopy
DGE: Dynamic glucose-enhanced MRI
FDG: Fluorodeoxyglucose
FEXI: Filter exchange imaging
FOV: Field of view
GBCA: Gadolinium-based contrast agent
GLUT: Glucose transporter
ICAM: Intercellular adhesion molecule
JAM: Junctional adhesion molecule
LRP: Low-density lipoprotein receptor–related protein
MCI: Mild cognitive impairment
MCT: Monocarboxylate transporter
MMP: Matrix metalloproteinase
MRI: Magnetic resonance imaging
MS: Multiple sclerosis
NMR: Nuclear magnetic resonance
PDGFR: Platelet-derived growth factor receptor
PECAM: Platelet endothelial cell adhesion molecule
PET: Positron emission tomography
RAGE: Receptor for advanced glycation end products
ROI: Region of interest
SPECT: Single photon emission computed tomography
SVD: Small vessel disease
TJ: Tight junction
WM: White matter
